# Low intensity psychological interventions for the treatment of feeding and eating disorders: a systematic review and meta-analysis

**DOI:** 10.1186/s40337-023-00775-2

**Published:** 2023-04-04

**Authors:** Emily Davey, Sophie D. Bennett, Rachel Bryant-Waugh, Nadia Micali, Andrea Takeda, Alexia Alexandrou, Roz Shafran

**Affiliations:** 1 UCL Great Ormond Street Institute of Child Health, University College London, 30 Guilford Street, London, WC1N 1EH UK; 2 Department of Child and Adolescent Psychiatry, Institute of Psychiatry, Psychology and Neuroscience, King’s College London, London, UK; 3 Maudsley Centre for Child and Adolescent Eating Disorders, South London and Maudsley NHS Foundation Trust, London, UK; 4 Department of Psychiatry, Faculty of Medicine, University of Geneva, Geneva, Switzerland; 5 Mental Health Services of the Capital Region of Denmark, Eating Disorders Research Unit, Ballerup Psychiatric Centre, Copenhagen, Denmark; 6 Barnet, Enfield and Haringey Mental Health NHS Trust, London, UK; 7 Winchester, UK

**Keywords:** Feeding and eating disorders, Low intensity psychological intervention, Self-help, Systematic review, Meta-analysis

## Abstract

**Background:**

Feeding and eating disorders are associated with significant illness burden and costs, yet access to evidence-based care is limited. Low intensity psychological interventions have the potential to increase such access.

**Methods:**

A systematic review and meta-analysis were conducted on the use of low intensity psychological interventions for the treatment of feeding and eating disorders. Studies comparing low intensity psychological interventions against high intensity therapies and non-eating disorder specific psychological interventions were included, as well as those with waiting list control arms. There were three primary outcomes: eating disorder psychopathology, diagnostic and statistical manual of mental disorders (DSM) severity specifier-related outcomes and rates of remission/recovery.

**Results:**

Thirty-three studies met the inclusion criteria, comprising 3665 participants, and 30 studies were included in the meta-analysis. Compared to high intensity therapies, low intensity psychological interventions were equivalent on reducing eating disorder psychopathology (*g* = − 0.13), more effective at improving DSM severity specifier-related outcomes (*g* = − 0.15), but less likely to achieve remission/recovery (risk ratio (RR) = 0.70). Low intensity psychological interventions were superior to non-eating disorder specific psychological interventions and waiting list controls across all three primary outcomes.

**Conclusion:**

Overall, findings suggest that low intensity psychological interventions can successfully treat eating disorder symptoms. Few potential moderators had a statistically significant effect on outcome. The number of studies for many comparisons was low and the methodological quality of the studies was poor, therefore results should be interpreted with caution. More research is needed to establish the effectiveness of low intensity psychological interventions for children and young people, as well as for individuals with anorexia nervosa, avoidant/restrictive food intake disorder, pica and rumination disorder.

**Supplementary Information:**

The online version contains supplementary material available at 10.1186/s40337-023-00775-2.

## Abstract (summary)

Feeding and eating disorders can significantly impair health and psychosocial functioning. However, demand for eating disorder services is greater than services’ ability to deliver effective treatment. Low intensity psychological interventions, which are brief in nature and require less therapist input than standard treatments, have the potential to bridge this demand-capacity gap. The current review examined the effectiveness of low intensity psychological interventions for the treatment of feeding and eating disorders. Overall, findings suggest that low intensity psychological interventions can successfully treat eating disorder symptoms, particularly binge eating-related symptoms. Given their relatively low costs and ease of accessibility, such interventions can help people to access treatment at a time when this is so desperately needed. More research is needed to determine the value of low intensity psychological interventions for children and adolescents, and people with feeding and eating disorders that are not characterised by recurrent binge eating, such as anorexia nervosa, ARFID, pica and rumination disorder.

**Supplementary Information:**

The online version contains supplementary material available at 10.1186/s40337-023-00775-2.

## Introduction

Eating disorders are common and disabling disorders that markedly impair physical health and disrupt psychosocial functioning [1]. They have high psychiatric and medical comorbidity, and one of the highest mortality rates among mental health disorders [2]. Eating disorders can substantially impact an individual’s health-related quality of life, and are associated with elevated healthcare utilisation and significant economic costs [3, 4]. Given the seriousness of these disorders and the associated illness burden and costs [5], there is a salient need for effective treatments.

Evidence-based, specialist psychological therapies have strong empirical support for the treatment of eating disorders [6, 7]. However, access to care for people with eating disorders has long been challenging [8], and has worsened since the COVID-19 pandemic [9]. The COVID-19 pandemic has adversely impacted individuals with eating disorders, with an increased incidence of first diagnosis [10], and deteriorating symptoms among those with pre-existing diagnoses [11]. This has imposed further strain on healthcare systems which were already over-stretched due to high staff vacancy rates and turnover, both in the UK [12, 13] and internationally [14]. This is concerning given that delays in receiving specialist treatment can increase the risk of chronicity and burden of illness [5]. While various geographical, financial and patient-associated barriers (e.g., fear of stigmatisation, ambivalence about change and poor mental health literacy) may contribute to this widening treatment gap [15], the reality is that the demand for eating disorder treatment far outweighs the availability of resources [16, 17].

Mental health professionals require specialised and intensive training to become competent in the delivery of evidence-based treatments for eating disorders [18], and the cost to implement face-to-face treatment is substantial [5]. Therefore, expanding the workforce of trained specialists to deliver conventional, face-to-face treatment is not a practical option [19, 20]. Instead, the treatment gap highlights the need to expand existing, evidence-based treatments to be delivered in ways that are more easily disseminable and affordable [17].

A central component of the extension of effective treatments to meet increased demand is the provision of ‘low intensity’ (LI) psychological interventions. LI psychological interventions are modified, brief versions of evidence-based therapies that can be delivered using a range of flexible delivery formats, such as bibliotherapy and digital platforms, and have a primary focus on teaching self-management skills to patients and/or their carers [21]. They require less therapeutic input than conventional treatments and can be delivered by practitioners who do not possess a core mental health professional qualification [22]. Thus, these interventions are considered low intensity from the provider’s perspective and do not reflect low engagement from the client. LI psychological interventions have the potential to reduce actual and perceived barriers to care [15], as well as unmet treatment needs, by providing more easily accessible services [23].

During the past decade, there has been a proliferation of LI psychological interventions for the treatment of eating disorders. In the UK, the National Institute for Health and Care Excellence (NICE) recommend cognitive behavioural therapy (CBT)-based guided self-help as the first line treatment for adults with bulimia nervosa (BN) and binge eating disorder (BED), as part of a stepped care treatment model [24]. However, it still remains unclear whether LI psychological interventions are effective for the broader range of feeding and eating disorders. The latest versions of the Diagnostic and Statistical Manual of Mental Disorders (DSM-5-TR; [25]) and the International Classification of Diseases and Related Health Problems (ICD; [26]) recognise six main feeding and eating disorders: anorexia nervosa (AN), BN, BED, avoidant/restrictive food intake disorder (ARFID), pica and rumination disorder; and a residual category: other specified feeding or eating disorder (OSFED), formerly known as eating disorder not otherwise specified (EDNOS).

It is important to understand the moderators that contribute to treatment outcome, as well as user satisfaction, in order to optimise how LI psychological interventions are developed and delivered, and to identify patients who are likely to benefit from such treatment [27]. People are more likely to adhere to treatment recommendations and ultimately benefit from improved clinical outcomes if they consider an intervention to be acceptable [28]. LI psychological interventions have traditionally been based on CBT principles but have more recently extended into other treatment modalities, such as dialectical behavioural therapy (DBT; [29]) and family-based treatment (FBT; [30]), so it is pertinent to explore the potential moderating effects of treatment modality on outcome. There has also been a shift in focus towards technology in health service delivery in recent years [31], so it is important to capture the relative effectiveness of internet-based interventions as well as interventions delivered via bibliotherapy.

Previous systematic review and meta-analyses (e.g., [32, 33]) evaluating the effects of LI psychological interventions for eating disorders have been limited in four important ways. First, they are rather narrow in focus, by aiming at specific patient groups (e.g., individuals with BN and BED [32]; young people [34]) and intervention formats (e.g., e-mental health [31]; self-help with guidance [33]). Second, some past reviews have focused solely on binge eating-related behavioural outcomes, such as binge eating frequency [32] and abstinence from binge eating [33], which precludes an exploration of effects for feeding and eating disorders where binge eating is not a key behavioural symptom. Third, the most recent review on guided self-help for adults only included randomised controlled trials (RCT) conducted to April 2016 [33], and a number of RCTs have been conducted since then that warrant inclusion in a review of the topic. Fourth, no previous review has used a published definition of LI psychological interventions. A broad, updated review is needed that includes all ages and interventions, and considers both active and inactive comparators in order to determine the specificity of any effects. Exploratory analyses to delineate the factors that may explain treatment outcomes, such as type of intervention (e.g., CBT, DBT) and mode of delivery (e.g., self-led, parent-led) are also warranted.

### Objectives

The present review and meta-analysis sought to systematically assess the evidence-base for the use of LI psychological interventions to treat feeding and eating disorders across young people and adults. Within this, the objectives were to:

- Investigate the efficacy of LI psychological interventions for feeding and eating disorders when compared to active (i.e., high intensity, therapist-delivered therapies, and non-eating disorder-specific psychological interventions) and inactive (e.g., waiting list) comparators at posttreatment and follow-up.
- Test whether these effects are moderated by certain participant (i.e., age, type of eating disorder) and intervention characteristics (i.e., type, format, mode of delivery, provision and type of guidance, qualification of guide).
- Assess the acceptability of these LI psychological interventions.

## Methods

The protocol for this systematic review and meta-analysis was prospectively registered with PROSPERO (CRD42022302956). It has been reported in accordance with the Preferred Reporting Items for Systematic Reviews and Meta-Analyses (PRISMA) 2020 guidelines ([35]; see Additional file 1).

### Eligibility criteria

#### Types of studies

Only RCTs were included to allow assessment of the highest-quality evidence available. Quasi-randomised trials (using alternate allocation) were excluded.

#### Types of participants

Participants meeting the DSM (versions III-R, IV, IV-TR, 5, 5-TR) or ICD (versions 9, 10, 11) diagnostic criteria for a feeding or eating disorder were eligible for inclusion. This included those with AN, BN, BED, ARFID, pica, rumination disorder, and OSFED (formerly EDNOS). A standardised assessment of feeding and eating disorder symptomatology was necessary to ascertain diagnoses with the DSM and ICD. There were no restrictions in terms of age (child, adolescent and adult), sex or gender.

#### Types of interventions

LI psychological interventions designed to treat feeding and eating disorders were included. For the purposes of this review, LI psychological interventions were defined as an intervention that (1) utilises self-help materials, (2) is 6 h or less of contact time (with each contact typically ≤ 30 min), and (3) any input is provided by practitioners or supporters who have been specifically trained to deliver the intervention [21]. The intervention had to be eating disorder-specific, and a stand-alone treatment to be included.

Studies were excluded if they evaluated LI psychological interventions integrated with another treatment, such as specialist face-to-face psychotherapy augmented with a LI psychological intervention. Studies were also excluded if the variable under experimental manipulation was not the LI psychological intervention, for example, a LI psychological intervention plus a smartphone app compared to a LI psychological intervention alone. We also excluded studies in which the LI psychological intervention was designed to prevent the onset of feeding and eating disorders. There were no restrictions on recruitment or treatment setting.

#### Types of comparators

Studies comparing a LI psychological intervention against a high intensity psychological treatment, a non-eating disorder specific psychological intervention, or a waiting list control condition were included in the review. Studies comparing two types of the same LI psychological intervention through different delivery formats (e.g., bibliotherapy vs online), and provision of guidance (guided vs unguided) were excluded. We also excluded studies which used a pharmacological treatment as the comparator.

#### Types of outcomes

Studies were included only if they reported core eating disorder outcomes at baseline and post-intervention at a minimum. Outcomes had to be assessed with standardised, well-validated measures in order to be comparable across studies. Studies were only included in the meta-analyses if statistics allowing for effect size estimation of core eating disorder outcomes (e.g., binge eating frequency, eating disorder-related attitudes) had been reported. Rates of remission/recovery were only extracted and analysed if definitions were outlined in the original manuscripts.

The primary outcomes were as follows:

1. *Eating disorder psychopathology* Operationalised using the most global measure of eating disorder psychopathology reported in each study. The Eating Disorder Examination (EDE), in interview or self-report questionnaire (EDE-Q) format, was prioritised for this analysis [36] due to it being the most widely used measure.
2. *DSM specifiers of severity* For BN, this was based on frequency of inappropriate compensatory behaviours (e.g., self-induced vomiting); for BED, on frequency of objective binge eating episodes; and for AN, on weight status (BMI; kg/m^2^). Due to concerns that BMI is not an optimal method to reflect nutritional status in adolescents [37], Expected Mean Body Weight (EBW) was also used in this analysis. ARFID, pica, rumination disorder and OSFED do not have severity specifiers.
3. *Remission/recovery* Definitions of remission/recovery varied across studies, with studies defining this variable as either (a) abstinence from binge eating and/or inappropriate compensatory behaviours over the past 28 days; (b) an EDE global score below one standard deviation of community norms; and (c) no longer meeting diagnostic criteria for an eating disorder. In one study, weight remission was defined as ≥ 95%EBW [38]. All four definitions were aggregated in the analyses.

The secondary outcomes were these core eating disorder outcomes at short (< 12 months) and long-term (≥ 12 months) follow-up, as well as drop-out rate and acceptability of the interventions. Qualitative results from measures of treatment acceptability were extracted where available.

### Information sources and search strategy

The main search strategy involved a search for published studies in the following databases: EMBASE, MEDLINE, PsycINFO, CINAHL and the Cochrane Central Register of Controlled Trials (CENTRAL). Grey literature searches were conducted in the ProQuest Dissertations and Theses Global repositories. Each database was searched from its year of inception to 27th January 2022, and then updated on 5th August 2022. Search terms, including MeSH terms, related to three concepts: (1) feeding and eating disorders; (2) low intensity psychological interventions; and (3) randomised controlled trials. Search terms were developed in collaboration with a librarian. See Additional file 2 for a full list of search terms used.

Reference lists of included studies and existing systematic reviews were searched for potentially relevant papers, and in-text citations of included studies were also screened. Additional literature was sought through personal contact with researchers in the area, and by hand searching relevant journals publishing on feeding and eating disorders, including the International Journal of Eating Disorders, European Eating Disorders Review and Journal of Eating Disorders. The search was restricted to publications in the English language.

### Study selection and data collection

Two reviewers (*ED* and *AA*) independently screened the titles and abstracts of all studies identified from the searches. The reviewers then independently examined the full texts and selected eligible RCTs. To aid full text screening, a screening tool using a hierarchical system was developed to determine on which ground a paper should be excluded. Disagreements were resolved through discussion or by consulting a third reviewer (*RS*). The systematic review software, Covidence, facilitated the screening process.

### Data extraction and management

Data extraction was carried out by one reviewer (*ED*), using a standardised data extraction form, and independently checked by a second reviewer (*AT*). Discrepancies were resolved through discussion. The following data were extracted from the eligible studies:

- Study identification details—first author, publication year, country.
- Study design characteristics—type of RCT, sample size, follow-up length.
- Participant characteristics—mean age, percentage female, criteria and assessment tool used to ascertain diagnosis.
- Intervention characteristics—type (e.g., CBT), format (e.g., bibliotherapy), mode (e.g., self-led), provision of guidance (guided or unguided), qualification of guide (if any).
- Comparator(s) characteristics—type (high intensity, non-eating disorder specific, waiting list).
- Outcome measures used, including definitions of remission/recovery.

We extracted means, standard deviations, and sample size at pre-intervention, post-intervention and at each follow-up thereafter (if any) in both the intervention and comparator groups. We also extracted remission/recovery data at post-treatment and follow-up. Wherever possible, data were extracted from intention-to-treat analyses, including the sample size at randomisation. Where completer analyses were conducted instead, we extracted the sample size of study completers to enable the weighting of the studies in the meta-analysis to be proportional to the amount of data contributed. If insufficient data were reported to meet the requirements for meta-analysis, missing data were requested from study authors to maximise the completeness of the meta-analytic review. If the contact attempts were unsuccessful, the papers were removed from the meta-analysis and included only in the narrative synthesis.

### Assessment of risk of bias in included studies

Risk of bias (RoB) was assessed using the criteria outlined in the Revised Cochrane Risk of Bias Tool for Randomised Trials (RoB version 2 [RoB 2]; [39]). Ten percent of studies were rated by a second independent rater (*AT*) and discrepancies were discussed until consensus was reached. RoB was assessed in the following domains: (1) randomisation process; (2) deviations from intended interventions; (3) missing outcome data; (4) measurement of the outcome; and (5) selection of the reported result. For cluster-RCTs, there is an additional domain for RoB arising from the timing of identification or recruitment of participants. RoB was assessed for each domain using a rating of low risk, high risk or some concerns. Consistent with previous meta-analyses (e.g., [40]), the impact of RoB was assessed by quantifying domain codes (low risk = 0, some concerns = 1, high risk = 2) and yielding a total RoB score ranging from 0 to 10 for each RCT and 0 to 12 for each cluster-RCT. We performed a meta-regression to examine the relationship between RoB and effect size, with the total RoB score entered as the dependent variable.

### Meta-analysis

#### Measurement of the treatment effect

The software program, Comprehensive Meta-Analysis version 3 [41], was used for computing and pooling effect sizes. In view of the considerable heterogeneity among the studies, a random effects model was adopted for all meta-analyses. Separate analyses were conducted for studies comparing against high intensity interventions, non-eating disorder-specific interventions and waiting list controls. For trials with more than one LI psychological intervention condition, effect sizes were calculated separately for each intervention.

For continuous outcomes of response (e.g., global EDE score), the effect size indicating the standardised mean difference (SMD) between the two groups at post-test (Hedges’ *g*) was calculated for each comparison. Hedges’ *g* was chosen as it adjusts for biases caused by small sample sizes [42]. A negative *g* favours LI psychological interventions over comparisons. SMDs were transformed into the Number Needed to Treat (NNT), using Kraemer and Kupfer’s [43] formulae. The NTT refers to the number of patients that have to be treated to achieve one additional positive outcome over a comparator. For dichotomous outcomes of response (e.g., abstinence from binge eating), the effect sizes were expressed in terms of the risk ratio (RR), otherwise known as relative risk. The RR is a ratio of the probabilities of achieving remission between two conditions. The RR was chosen because it is easier to interpret than the odds ratio [42]. An RR greater than 1 favours LI psychological interventions over comparisons. We recalculated remission and recovery rates for the intent-to-treat analyses using the number of randomised participants as the denominator of the proportion of remission/recovery; as such, remission and recovery rates in this review may differ from those reported in the original manuscripts. 95% Confidence Intervals (CIs) were calculated for each outcome. Where two or more measures were used per outcome, they were combined and the pooled effect size was calculated so that only one effect size per study was included in the analysis.

A series of subgroup analyses were performed according to the mixed effects model. In this model, studies within subgroups are pooled using a random effects model, while tests for significant differences between subgroups are conducted within the fixed effects model [44]. For continuous variables (e.g., age), meta-regression analyses were used to examine whether there was a significant relationship between the continuous variable and the effect size, as indicated by a regression coefficient (*Z* value) and associated *p* values. We aimed to explore the potential moderating effects of the following variables:

- Participant age.
- Type of eating disorder—[BN, BED, AN, ARFID, pica, rumination disorder, OSFED or mixed (to include transdiagnostic studies)].
- Treatment modality (e.g., CBT, DBT).
- Format of intervention (e.g., bibliotherapy, online).
- Mode of delivery (e.g., self-led, parent-led).
- Provision of guidance (guided vs unguided).
- Type of guidance (e.g., email, telephone).
- Qualification of guide (non-specialist, mental health specialist, eating disorder/CBT specialist).

#### Assessment of heterogeneity

Statistical heterogeneity was examined using Cochran’s *Q* and *I*^2^ statistics [45]. A significant *Q* statistic indicates varying effect sizes across studies as well as sample or methodological differences that may contribute to variance. The *I*^2^ statistic assesses the percentage of variability due to heterogeneity rather than to random error. A value of 0% indicates no observed heterogeneity, whereas scores of 25%, 50% and 75% indicate low, moderate and high heterogeneity, respectively.

#### Assessment of publication bias

Publication bias was examined through visual inspection of a funnel plot, and by using Egger’s regression intercept to test funnel plot asymmetry [46]. We also used Duval and Tweedie's [47] trim-and-fill procedure, which estimates the number of studies that have to be removed to make the funnel plot symmetrical, and then imputes an estimated effect size after publication bias has been taken into account.

## Results

### Narrative synthesis

#### Results of search

**Fig. 1:**
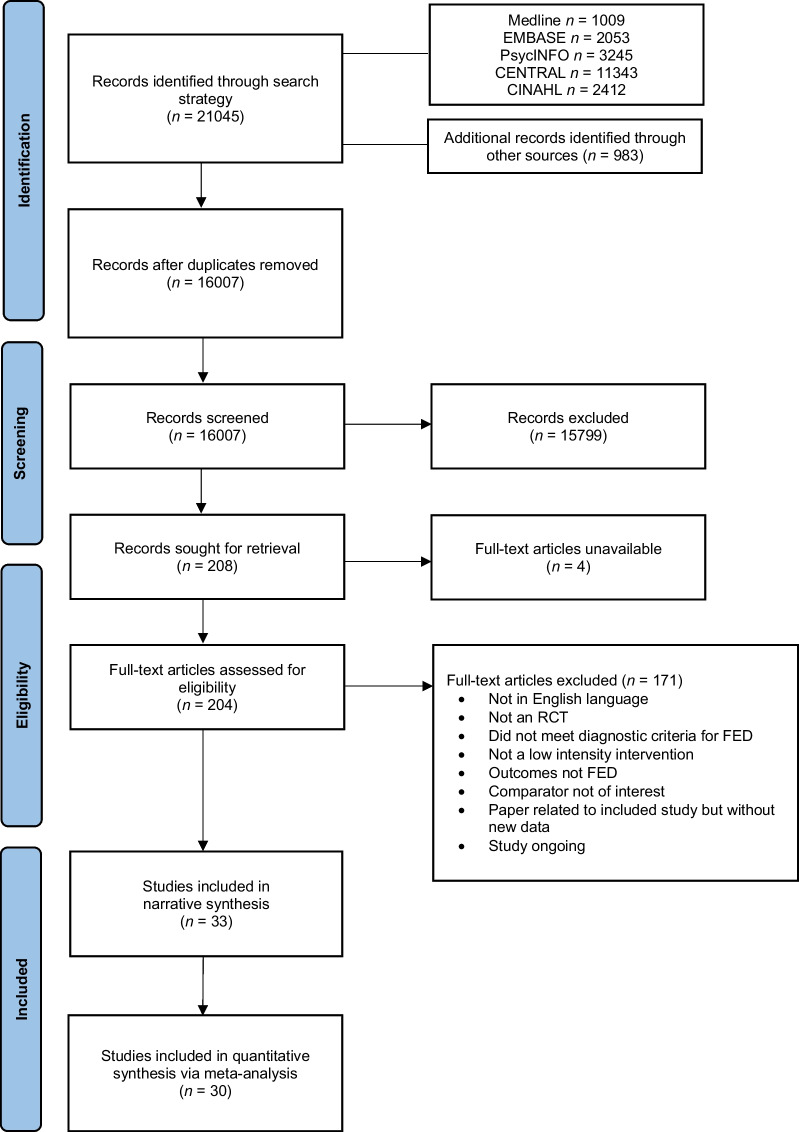
PRISMA flow diagram of study selection

As illustrated in the PRISMA flow diagram (see Fig. 1), the search strategy yielded 16,007 articles after the removal of duplicates. Following title and abstract screening, a total of 204 full-text papers were retrieved, of which 171 were excluded because they did not meet the inclusion criteria. Thirty-three RCTs met inclusion criteria for the narrative synthesis, including one cluster-RCT and seven pilot/feasibility RCTs. Nineteen of the studies included in the current investigation were not available in the most recent meta-analyses conducted on this topic [33].

#### Sample and study characteristics (Table 1)

**Table 1:** Characteristics of included studies

Study	Country	Participants	Mean age, years (SD)	Outcome measures	Low intensity psychological intervention(s)	Comparator(s)	RoB (total/10)
Jenkins et al.* [54]	UK	*N* = 126; BN, BED, OSFED; 92.8% female; adults	30.5 (10.6)	EDE-Q	CBT GSH-F and CBT GSH-E (overcoming binge eating manual); 9 × 25 min sessions; clinical psychologists, qualified nurses with mental health experience (one of who had advanced training in CBT), paraprofessionals	Delayed treatment control	+ − + + − (6)
Lock et al. [38]	USA and Canada	*N* = 40; AN, 85% female, adolescents	14.9 (1.81)	%EBW; BMI; EDE	FBT GSH; 12 × 30 min sessions; PhD psychologists, an MD psychiatrist or licensed social workers (all experienced in FBT)	FBT via videoconferencing; 15 × 60 min sessions; Ph.D. psychologists, an MD psychiatrist or licensed social workers (all experienced in FBT)	– – + + –(4)
Wyssen et al. [55]	Switzerland	*N* = 63, BED, 87% female, adults	37.2 (10.4)	Mini-DIPS; EDE-Q; WBQ	Internet-based CBT GSH (BED-Online); 8 sessions; psychotherapists and psychologists in postgraduate training of psychotherapy	Waiting list control	+ – + + – (6)
Carter et al. [56]	Canada	*N* = 71; BED; 93% female, adults	40.7 (11.5)	EDE	DBT GSH and DBT USH (the DBT solution for emotional eating manual); DBT GSH 6 × 30 min; DBT GSH clinical psychology graduates	Self-esteem USH (self-esteem: a proven program of cognitive techniques for assessing, improving, and maintaining your self-esteem)	○ − + + ○ (6)
Fitzsimmons-Craft et al. [57]	USA	*N* = 690; BN, BED, purging disorder, unspecified feeding or eating disorder; 100% female; adults	22.1 (4.9)	EDE-Q	Digital CBT GSH (SB-ED); 2 × 20 min optional telephone calls and asynchronous text-based support (~ 16 messages per participant); psychology doctoral students, social work master’s students, study staff and postdoctoral fellows	Referral to usual care	− + – + + ○ (7/12)
Hildebrandt et al. [58]	USA	*N* = 225; BN, BED; 75% female; adults	41.2 (9.9)	EDE-Q	CBT GSH plus Noom Monitor; 8 × 25 min sessions; certified health coaches	Standard care	− − + + − (4)
Peterson et al. [59]	USA	*N* = 112; BED; 82.1% female; adults	39.7 (13.4)	EDE	CBT GSH (overcoming binge eating manual); 10 × 30 min sessions; master’s level clinician without specialisation in eating disorders	ICAT-BED; 21 × 50 min sessions; doctoral-level psychologists and graduate students	− − + + − (4)
Cachelin et al. [60]	USA	*N* = 40; BED; 100% female; adults	27.0 (8.9)	EDE	CBT GSH (culturally adapted overcoming binge eating manual); 8 × 25 min sessions; graduate- and senior-level undergraduate psychology students	Waiting list control	○ − + + ○ (6)
Green et al. [61]	USA	*N* = 82; AN, BN, BED, OSFED; 100% female; adults	26.1 (6.1)	EDE-Q	Online dissonance-based program (the Body Project)	Waiting list control	○ − + + ○ (6)
de Zwaan et al. [62]	Germany	*N* = 178; BED; 87.6% female; adults	43.2 (12.3)	EDE	Internet-based CBT GSH; 2 × 90 min sessions pre- and post-treatment, and weekly email contacts over a 4-month period; coaches	Face-to-face CBT; 20 × 50 min sessions; CBT therapists	− − + + − (4)
Duarte et al. [49]	Portugal	*N* = 22; BED; 100% female; adults	37.7 (7.5)	EDE; BES	CARE USH (manual)	Waiting list control	○ + + + ○ (8)
Strandskov et al. [50]	Sweden	*N* = 92; BN, EDNOS; 96.7% female; adults	29.1 (9.7)	EDE-Q	Online ACT-influenced CBT program; written feedback on website and phone calls (~ 15 min per week for 8 weeks); clinical psychology master’s students	Waiting list control	○ − + + ○ (6)
Kelly and Carter [63]	Canada	*N* = 31; BED; 83% female; adults	45.0 (15)	EDE-Q	Behavioural strategies USH and Self-compassion USH	Waiting list control	○ − + + ○ (6)
ter Huurne et al. [64]	Netherlands	*N* = 213; BN, BED, EDNOS; 100% female; adults	39.4 (11.6)	EDE-Q	Web-based CBT program (Look at your eating); asynchronous internet-based contact; therapists with a bachelor’s degree in nursing or social work or a master’s degree in psychology	Waiting list control	− − + + − (4)
Grilo et al. [65]	USA	*N* = 90; BED; 79% female; adults	45.8 (11.0)	EDE; EDE-Q	CBT USH (overcoming binge eating manual)	Usual care	– − − + ○ (3)
Masson et al. [66]	Canada	*N* = 60; BED; 88.3% female; adults	42.8 (10.5)	EDE; EDE-Q	DBT GSH (DBT for binge eating manual); 6 × 20 min sessions; researcher	Waiting list control	+ − + + ○ (7)
Carrard et al. [67]	Switzerland	*N* = 74, BED; 100% female; adults	36.1 (11.4)	EDE-Q; EDI-2; TFEQ	Internet-based CBT GSH (online programme adapted from Overcoming Binge Eating); weekly e-mail contact; psychologists	Waiting list control	+ − + + ○ (7)
Sánchez-Ortiz et al. [68]	UK	*N* = 76; BN, EDNOS; 98.7% female; adults	23.9 (5.9)	EDE	Internet-based CBT (overcoming bulimia online), weekly email contact; CBT therapists with eating disorder experience	Delayed treatment control	− − + + ○ (5)
Traviss et al. [69]	UK	*N* = 81; BED; 97% female; adults	36.9 (11.9)	EDE-Q	CBT GSH (working to overcome eating difficulties manual); one 1-h introductory session and 6 × 1 h sessions; trained mental health professionals	Waiting list control	○ − + + ○ (6)
Striegel-Moore et al. [70]	USA	*N* = 123; BN, BED; 91.9% female; adults	37.2 (7.8)	EDE	CBT GSH (overcoming binge eating manual); one 1-h introductory session and 7 × 25 min sessions; master’s level therapists with no familiarity with eating disorders or treating binge eating	Treatment as usual	○ − + +○ (6)
Wilson et al. [71]	USA	*N* = 205; BED; 85.4% female; adults	48.3	EDE	CBT GSH (overcoming binge eating manual); one 1-h introductory session and 9 × 25 min sessions; first- or second-year graduate students with no experience in CBTgsh or treating BED	IPT; one 2-h introductory session and 19 × 60 min sessions and BWL 20 × 50 min sessions; IPT doctoral-level therapists; BWL not included in meta-analysis	○ − + + ○ (6)
Schmidt et al. [72]	UK	*N* = 97; BN, EDNOS; 96.9% female; adults	27.1 (7.6)	EDE	CD-ROM-based CBT programme (Overcoming Bulimia)	Waiting list control	– − − + ○ (3)
Steele and Wade [73]	Australia	*N* = 48; BN, EDNOS, 98.9% female; adults	26.0 (5.83)	EDE	CBT GSH (bulimia nervosa and binge-eating; 8 × 40 min sessions; postgraduate psychology students	Placebo GSH (Mindfulness-Based Cognitive Therapy for Depression); 8 × 40 min sessions; postgraduate Psychology students Perfectionism GSH (When Perfect Isn’t Good Enough) not included in meta-analysis	○ − + + ○ (6)
Ljotsson et al. [74]	Sweden	*N* = 73; BN, BED; 94.2% female; adults	34.6 (10.4)	EDE; EDI-2	CBT GSH (Swedish translation of overcoming binge eating manual); weekly email contact; graduate psychology students	Waiting list control	○ − + + ○ (6)
Shapiro et al. [75]	USA	*N* = 66; BED; 92.4% female; adults	39.6 (11.7)	QEWP-R; BES	CD-ROM-based CBT programme (based on cognitive-behavioural treatment for healthy weight control); one brief telephone contact per week; research assistant	Group CBT (based on cognitive-behavioural treatment for healthy eating and weight control) and waiting list control; group CBT 10 × 90 min group sessions; group CBT Ph.D. level clinical psychologist	○ − + + ○ (6)
Banasiak et al. [76]	Australia	*N* = 109; BN, 100% female, adults	28.9 (8.5)	EDE	CBT GSH (Bulimia Nervosa and Binge-Eating: A Guide to Recovery manual); one 1-h introductory session and 9 × 30 min sessions; GPs with no postgraduate or specialist qualification is psychology or psychiatry	Delayed treatment control	– − + + ○ (5)
Grilo and Masheb [77]	USA	*N* = 90; BED; 79% female; adults	46.3 (9.0)	EDE-Q; TFEQ	CBT GSH (overcoming binge eating manual); 6 × 20 min sessions; doctoral research-clinicians trained in CBT and BED	BWL GSH (LEARN Program for Weight Management manual) 6 × 20 min sessions; doctoral research-clinicians trained in CBT and BED; and no treatment manual control not included in meta-analysis	– − + + ○ (5)
Bailer et al. [48]	Austria	*N* = 81; BN; adults	23.8 (4.5)	EB-IV; EDQ; EDI	CBT GSH (German version of Getting Better Bite by Bite); 18 × 20 min sessions; first- and second-year residents in psychiatry with no experienced with eating disorders or formal psychotherapy training	Group CBT; 18 × 90 min group sessions; experienced therapists	○ − + + ○ (6)
Carter et al.* [78]	Canada	*N* = 72; BED; 100% female; adults	39.7 (10)	EDE; EDE-Q; EDI	CBT USH (overcoming binge eating manual)	Nonspecific USH (self-assertion for women manual) and waiting list control	– − + + ○ (5)
Durand and King [79]	UK	*N* = 68; BN; 100% female; adults	26.4 (5.85)	BITE; EDE	CBT GSH (Bulimia Nervosa: a guide to recovery manual); regular contact; general practitioners (GPs)	Specialist clinic treatment (combination of CBT and IPT); weekly or fortnightly session; psychiatrists, psychologists, nurse specialists and dietitians	– − + + ○ (5)
Palmer et al. [80]	UK	*N* = 121; BN; 96.7% female; adults	26.9 (8.4)	EDE	CBT GSH-F, CBT GSH-T and CBT SH-MG (overcoming binge eating manual); CBT GSH-F and CBT GSH-T 4 × 30 min sessions, and CBT SH-MG one brief session; nurse therapists experienced in eating disorder treatment	Waiting list control	– − + + ○ (5)
Carter and Fairburn [81]	UK	*N* = 72; BED; 100% female; adults	39.7 (1)	EDE; EDE-Q	CBT GSH and CBT USH (Overcoming Binge Eating manual); CBT GSH 8 × 25 min sessions; non-specialist therapists working in primary care	Waiting list control	– − + + ○ (5)
Treasure et al.* [82]	UK	*N* = 81; BN; 100% female; adults	25.8 (4.18)	EDI; BITE	CBT USH (Getting Better Bite by Bite manual)	CBT and Waiting list control; CBT 16 sessions; CBT therapist	○ + + + ○ (8)

*AN* anorexia nervosa, *BED* binge eating disorder, *BES* binge eating scale, *BITE* bulimic investigatory test Edinburgh, *BMI* body mass index, *BN* bulimia nervosa, *BWL* behavioural weight loss, *CARE* compassionate attention and regulation of eating behaviour, *CBT* cognitive behavioural therapy, *DBT* dialectical behaviour therapy, *EB-IV* eating behaviour-IV, *EDE* eating disorder examination, *EDE-Q* eating disorder examination questionnaire, *EDI* eating disorder inventory, *EDNOS* eating disorder not otherwise specified, *EDQ* eating disorder questionnaire, *FBT* family-based treatment, *GSH* guided self-help, *GSH-E* guided self-help with email guidance, *GSH-F* guided self-help with face-to-face guidance, *GSH-T* guided self-help with telephone guidance, *ICAT* integrative cognitive-affective therapy, *IPT* interpersonal psychotherapy, *Mini-DIPS* diagnostic interview for mental disorders, short version, *OSFED* other specified feeding or eating disorder, *QEWP-R* questionnaire on eating and weight patterns-revised, *SB-ED* student bodies-eating disorders, *SH-MG* self-help with minimal guidance, *TFEQ* three-factor eating questionnaire, *USH* unguided self-help, *WBQ* weekly binges questionnaire, *%EDW* expected mean body weight, *RoB* risk of bias, +  high risk of bias, ○ some concerns, − low risk of bias, for each of the categories considered: the randomisation process, deviations from the intended intervention, missing outcome data, measurement of the outcome and selection of the reported result

Studies with an asterisk* were included in the narrative synthesis but not in the meta-analysis

The included studies encompassed 3665 individuals with eating disorders. The majority of studies included participants aged 18 years or older; only one study focused on adolescents (i.e., aged 12–18 years; [38]). The studies were predominantly comprised of females, with ten studies having exclusively female participants. Participant gender was not stated in one study [48]. The majority of studies focused on participants with BED (*n* = 15) and BN (*n* = 5), and one study focused on participants with AN [38]. Twelve of the studies included ‘mixed’ samples with a range of eating disorder diagnoses. No studies included participants with ARFID, pica or rumination disorder.

Across the 33 included studies, 39 LI psychological interventions to treat eating disorders were investigated. The most commonly studied treatment modality was CBT (*n* = 31). Other treatment modalities included Compassion-Focused Therapy (*n* = 1), DBT (*n* = 3), FBT (*n* = 1) and a dissonance-based program (*n* = 1). Two studies used a LI psychological intervention that combined elements from multiple treatment modalities, such as Compassion Attention and Regulation of Eating Behaviour [49] and Acceptance and Commitment Therapy (ACT)-influenced CBT [50]. The majority of studies delivered the LI psychological intervention with a manual or book via bibliotherapy (*n* = 28), nine delivered the intervention using an online platform and two studies used a CD-ROM.

Of the 33 RCTs, eight studies compared a LI psychological intervention against a high intensity psychological intervention, nine against a non-eating disorder-specific psychological intervention and 21 against a waiting list control group. High intensity therapies included group CBT (*n* = 2), individual CBT (*n* = 2), FBT (*n* = 1), Interpersonal Psychotherapy (IPT; *n* = 1), Integrative Cognitive-Affective Therapy (ICAT; *n* = 1), and a specialist outpatient treatment which combined CBT and IPT (*n* = 1). Non-eating disorder-specific psychological interventions included self-esteem unguided self-help (*n* = 1), perfectionism guided self-help (*n* = 1), behavioural weight loss guided self-help (*n* = 1), mindfulness-based CBT guided self-help for depression (*n* = 1), a self-assertion manual (*n* = 1), behavioural weight loss treatment (*n* = 1), and usual care or referral to usual care (*n* = 3).

Although a range of measures were considered appropriate to quantify eating disorder outcomes, most studies administered the EDE [51] or EDE-Q [36] to assess eating disorder psychopathology. Alternative outcome measures included in these analyses were the Eating Disorder Inventory (EDI; [52]) and the Binge Eating Scale (BDI; [53]). Full sample and study characteristics are outlined in Table 1.

### Risk of bias within randomised controlled trials

Table 1 summarises the RoB across all domains for each study. All studies were considered to be high RoB for ‘measurement of the outcome’ due to the inability of blinding participants to treatment condition and the use of self-report measures. As the default overall judgment for each study is high RoB when one of the domains is judged at high risk [39], all studies were rated as high RoB. The median RoB score was 6 out of 10 (range = 3–8) and 25 studies had a total RoB score of ≥ 5. The one cluster-RCT included in the review [57] had a total RoB score of seven (out of 12). Fifteen of the 33 studies performed well regarding the conduct and reporting of the randomisation process. Most studies conducted intent-to-treat (or modified intent-to-treat) analyses; however, two studies conducted completer analyses only. The domain ‘missing outcome data’ was frequently rated as being high RoB across studies (n = 31) due to a significant proportion of missing data (> 5%), as a result of high dropout and/or reasons suggesting attrition may be due to mental health status. All studies consistently measured relevant outcomes across the intervention and comparator groups, however, as previously stated, they all employed self-report measures. Only 9 of the 33 RCTs had a published or prospectively registered protocol, meaning it was not possible to determine whether the outcome analyses and reporting was consistent with the authors’ prespecified protocol.

### Treatment acceptability

Only half (*n* = 16) of the studies reported on treatment acceptability, but among those that did, findings suggest that LI psychological interventions were regarded acceptable, as indexed by self-reported satisfaction displayed in Table 2. Some studies demonstrated lower acceptability for LI interventions when compared to high intensity, face-to-face treatment [57]. However, Lock et al. [38] found similar acceptability rates between FBT delivered via guided self-help and high intensity FBT delivered via videoconferencing.

**Table 2:** Self-reported satisfaction with low intensity psychological interventions across studies

Study	Measure	Main findings
Jenkins et al.* [54]	–	–
Lock et al. [38]	Therapy suitability and patient expectancy (TSPE)	Parents reported the intervention as both suitable and acceptable. At the end of session 1, parents’ ratings on the TSPE were as follows: Suitability of the treatment (M = 7.9; SD = 2.0) Expectations of therapy (M = 7.4; SD = 1.8)
	The Helping Alliance Questionnaire (HAQ; De Weert-Van Oene et al., 1999)	Parents rated the following domains at session 1 and session 8: Improvement scores rose from M = 2.6 (SD = 0.9) to M = 3.8 (SD = 0.9) Helpfulness subscale rose from M = 4.8 (SD = 4.8) to M = 7.8 (SD = 4.4) Cooperation subscale M = 11.9 (SD = 5.7) to M = 12.4 (SD = 4.8)
Wyssen et al. [55]	Custom treatment satisfaction scale	Treatment satisfaction of completers was high with a mean value of 8.3/10 (SD = 1.5) Reasons for discontinuation included: Burden/strain (6.3%) Dissatisfaction with the program (4.8%) Lack of time (4.8%) Lack of motivation (4.8%) Switch to another treatment (1.6%)
Carter et al. [56]	Custom suitability and effectiveness scale	Participants were generally very satisfied with both the guided and unguided self-help versions of the intervention Guided self-help: suitability (M = 88.8/100; SD = 15.2) and effectiveness (M = 77.3/100; SD = 17.8) Unguided self-help: suitability (M = 75.3/100; SD = 23.2) and effectiveness (M = 66.8/100; SD = 19.3)
Fitzsimmons-Craft et al. [57]	–	–
Hildebrandt et al. [58]	–	–
Peterson et al. [59]	Therapy suitability and patient expectancy (TSPE)	Participants were generally satisfied with the intervention, with a mean score of 8.7/10 (SD = 1.7) for treatment suitability and 8.3 (SD = 1.5) in terms of expectations for success
Cachelin et al. [60]	Client satisfaction questionnaire (Attkisson and Zwick, 1982)	Participants who completed the intervention (n = 15) reported a high level of satisfaction with the programme. Mean score 30.5/32 (SD = 1.91; range 26–32)
Green et al. [61]	–	–
de Zwaan et al. [62]	–	-
Duarte et al. [49]	Custom feedback on intervention questionnaire	Most participants reported that the practices were very useful and rated the materials within the programme as very important
Strandskov et al. [50]	–	–
Kelly and Carter [63]	The credibility/expectancy questionnaire (Devilly and Korkovec, 2000)	Participants were fairly satisfied with both the behavioural strategies intervention and self-compassion intervention Behavioural strategies: intervention credibility (M = 7.0/10; SD = 1.2) and binge reduction expectancy (M = 71.8%; SD = 20.4) Self-compassion intervention: intervention credibility (M = 7.2/10; SD = 1.3) and binge reduction expectancy (M = 69.1%; SD = 19.7)
ter Huurne et al. [64]	Custom treatment acceptability scale	Participants were satisfied with both the intervention and their therapist. Most participants evaluated the intervention as rather (46%, 42/91) or very (35%, 32/91) useful. On average, participants rated the intervention with a 7.6/10 (SD = 1.3) and their therapist with an 8.1 (SD = 1.0) The majority of participants considered the online contact to be (very) pleasant (77%; 70/91), personal (60%; 55/91) and safe (92%; 84/91). Almost all participants said that the support of the therapist added value and identified the therapeutic support as one of the most valuable and important components of the treatment Some participants missed other forms of contact (e.g., face-to-face or via telephone) Reasons for dropping out or stopping the intervention prematurely included: Personal reasons or problems (33%; e.g., lack of time, psychological problems, lack of motivation) Treatment content/protocol (29%; e.g., eating diary annoying/too time consuming, assignments not supportive, not enough attention for weight loss) Online method (21%; e.g., lack of contact, too open-ended)
Grilo et al. [65]	–	–
Masson et al. [66]	–	–
Carrard et al. [67]	Custom satisfaction with programme questionnaire	No data reported, but states that the programme was well accepted by individuals with BED who are seeking treatment
Sánchez-Ortiz et al. [68]	–	–
Traviss et al. [69]	–	–
Striegel-Moore et al. [70]	Custom acceptability and treatment expectancies Scale	Participants found the intervention to be suitable (M = 4.2/5; SD = 0.7) and were reasonably confident that the treatment would be successful (M = 3.8/5; SD = 0.8)
Wilson et al. [71]	Custom treatment expectations and treatment suitability Scale	Participants were generally satisfied with the intervention, rating treatment suitability as 7.6/10 (SD = 2.1) and likely effectiveness 7.5 (SD = 2.2)
Schmidt et al. [72]	–	–
Steele and Wade [73]	–	–
Ljotsson et al. [74]	–	–
Shapiro et al. [75]	–	–
Banasiak et al. [76]	Custom attitudes towards treatment scale	Attitudes towards treatment scores were favourable. Mean Satisfaction with Treatment score was 6.89/10 (SD = 2.46), Satisfaction with GP score was 6.25/10 (SD = 3.20), Satisfaction with Treatment Outcome score was 5.93 (SD = 2.51) and Treatment Credibility score was 8.36 (SD = 2.24)
Grilo and Masheb [77]	Custom treatment expectations and treatment suitability Scale	Participants rated the extent to which the treatment was ‘logical’ as high (M = 8.8/10; SD = 1.3)
Bailer et al. [48]	–	–
Carter et al.* [78]	Custom suitability and likely effectiveness of treatment scale	Participants reported moderate levels of satisfaction with the intervention Suitability: M = 6.7/10; SD = 2.2 Expected effectiveness: M = 4.8/10; SD = 2.5
Durand and King [79]	Custom satisfaction with treatment questionnaire	Most participants found some aspects of the self-help programme helpful. The intervention was praised for: Behaviourally-focused early stages Having a structure to follow Having someone to talk to Criticisms included: Time consuming and discipline Time constraints of GP affected their GP’s ability to help them Attending the clinic because of work commitments Proposed improvements to self-help programme: More frequent/longer appointments GP training More active participation on the part of therapists Involvements of other professionals Meeting other patients with similar problems
Palmer et al. [80]	–	–
Carter and Fairburn [81]	Custom suitability and likely effectiveness of treatment scale	Participants rated both the guided and unguided self-help versions of the intervention to be highly credible Guided self-help: suitability (M = 7.3/10; SD = 2.7) and likely effectiveness (M = 8.6/10; SD = 1.8) Unguided self-help: suitability (M = 7.0/10; SD = 1.7) and likely effectiveness (M = 8.1/10; SD = 1.5)
Treasure et al.* [82]	–	–

*Studies with an asterisk were included in the narrative synthesis but not in the meta-analysis

### Study attrition rates

The attrition rate was calculated as the proportion of randomised participants who did not have post-treatment data. 31 studies provided information about attrition at post-treatment; the mean attrition rate across these studies was 21.6%, ranging from 0% [65] to 44.4% [69]. There were two studies that did not provide sufficient data to calculate total study attrition rates. Jenkins et al. [54] reported a drop-out rate of 36.9% in the self-help with face-to-face guidance group and a significantly higher drop-out rate of 67.9% in the self-help with email guidance group. However, the proportion of waiting list participants who dropped out during the treatment phase was not stated. Treasure et al. [82] provided details regarding the number of randomised participants who dropped out during the treatment phase (*n* = 29); however, the total number of randomised participants was not stated and only completer analyses were conducted. Some studies that compared a LI intervention to a high intensity intervention reported a higher drop-out rate among those who received the LI intervention [59, 62, 71]. However, Bailer et al. [48] found the drop-out rate did not differ between their guided self-help condition and high intensity, group CBT condition. Further details on attrition rates for each study can be found in Additional file 3.

### Meta-analysis

Thirty studies provided sufficient data to be included in the meta-analysis. Separate analyses are presented for studies comparing against a high intensity psychological intervention (Table 3), a non-eating disorder-specific psychological intervention (Table 4) and a waiting list control condition (Table 5). For continuous outcomes (i.e., eating disorder psychopathology and DSM severity specifiers), an effect size (*g*) below 0 favours LI psychological interventions. For dichotomous outcomes (i.e., remission and recovery rates), an effect size (RR) above 1 favours LI psychological interventions.

**Table 3:** Meta-analysis results for studies comparing a low intensity psychological intervention against a high intensity psychological intervention

	Ncomp	ES	95%CI	*Z*	*I*^*2*^	*p*	NNT	*Q* (*p*)
Eating disorder psychopathology (*g*)	7	− 0.13	− 0.30 to 0.04	− 1.51	17.83	.13	13.51	7.30 (0.29)
Only studies with a total risk of bias score of ≤ 4	3	− 0.06	− 0.28 to 0.16	− 0.53	< .001	.60	29.41	0.13 (0.94)
Effect at < 12 months follow-up	4	− 0.20	− 0.40 to − 0.01	− 2.02	< .001	.04*	8.93	0.71 (0.87)
DSM severity specifier (*g*)	7	− 0.15	− 0.31 to 0.00	− 1.99	< .001	< .05*	11.11	3.35 (0.76)
Only studies with a total risk of bias score of ≤ 4	3	− 0.16	− 0.38 to 0.06	− 1.44	< .001	.15	11.11	1.88 (0.39)
Effect at < 12 months follow-up	4	− 0.11	− 0.32 to 0.10	− 1.05	9.10	.30	16.13	3.30 (0.35)
Effect at ≥ 12 months follow-up	3	− 0.12	− 0.32 to 0.08	− 1.22	< .001	.22	14.71	0.69 (0.71)
Remission/recovery (RR)	5	0.70	0.56 to 0.87	− 3.19	< .001	< .01**		1.94 (0.75)
Only studies with a total risk of bias score of ≤ 4	3	0.68	0.54 to 0.86	− 3.30	< .001	< .01**		0.85 (0.55)
Effect at < 12 months follow-up	4	0.81	0.64 to 1.01	− 1.84	< .001	.07		0.73 (0.87)

For hedges’ *g,* negative values favour low intensity psychological intervention. For risk ratio, values > 1 favour low intensity psychological intervention

*Ncomp* number of comparisons, *ES* effect size

*p ≤ .05; **p ≤ .01

**Table 4:** Meta-analysis results for studies comparing a low intensity psychological intervention against a non-eating disorder specific intervention

	Ncomp	ES	95%CI	*Z*	*I*^*2*^	*p*	NNT	*Q* (*p*)
Eating disorder psychopathology (*g*)	5	− 0.35	− 0.49 to − 0.22	− 5.11	< .001	< .01**	5.10	0.09 (> .99)
Effect at < 12 months follow-up	3	− 0.31	− 0.66 to 0.04	− 1.76	< .001	.73	5.75	0.65 (0.73)
DSM severity specifier (*g*)	6	− 0.22	− 0.34 to − 0.10	− 3.66	< .001	< .01**	8.06	3.20 (0.67)
Only studies with a total risk of bias score of ≤ 4	2	− 0.39	− 0.62 to − 0.15	− 3.17	< .001	< .01**	4.59	0.15 (0.70)
Effect at < 12 months follow-up	3	− 0.17	− 0.39 to 0.04	− 1.56	< .001	.12	10.42	0.48 (0.79)
Remission/recovery (RR)	7	1.47	1.13 to 1.92	2.87	15.48	< .01**		7.10 (0.31)
Only studies with a total risk of bias score of ≤ 4	2	1.63	0.99 to 2.68	1.93	< .001	.05*		0.71 (0.40)
Effect at < 12 months follow-up	4	1.93	1.48 to 0.53	4.81	1.61	< .01**		3.05 (0.38)

For hedges’ *g*, negative values favour low intensity psychological intervention. For risk ratio, values > 1 favour low intensity psychological intervention

*Ncomp* number of comparisons, *ES* effect size

*p ≤ .05; **p ≤ .01

**Table 5:** Meta-analysis results for studies comparing a low intensity psychological intervention against waiting list controls

	Ncomp	ES	95%CI	*Z*	*I*^*2*^	*p*	NNT	*Q* (*p*)
Eating disorder psychopathology (*g*)	15	− 0.68	− 0.90 to − 0.46	− 6.05	66.57	< .01**	2.70	41.88 (< .01)
Only studies with a total risk of bias score of ≤ 4	2	− 0.24	− 0.56 to 0.07	− 1.54	43.69	.13	7.46	1.78 (0.18)
DSM severity specifier (*g*)	14	− 0.60	− 0.74 to − 0.45	− 8.05	< .001	< .01**	3.05	8.77 (0.79)
Remission/recovery (RR)	11	3.01	1.93 to 4.69	4.87	< .001	< .01**		7.55 (0.67)

For hedges’ *g*, negative values favour low intensity psychological intervention. For risk ratio, values > 1 favour low intensity psychological intervention

*Ncomp* number of comparisons, *ES* effect size

*p ≤ .05; **p ≤ .01

***p-values are provided in the column titled *p*, and the *p*-values which reached significance have "*"/"**" after them

#### Low intensity psychological interventions vs high intensity psychological interventions.

Effect size data for the seven studies comparing against a high intensity psychological intervention can be found in Table 3 (7 comparisons). Forest plots of effect sizes on each primary outcome for studies comparing against a high intensity psychological intervention are presented in Additional file 4.1. Effect size data for each subgroup analyses are displayed in Additional file 4.2. See Additional file 4.3 for funnel plots examining publication bias.

For eating disorder psychopathology, the pooled between-group effect size (*g*) at post-treatment was − 0.13 (95% CI [− 0.30, 0.04], *p* = 0.13; NNT = 13.51), suggesting low and high intensity psychological interventions were equally efficacious at reducing eating disorder psychopathology. At short-term (< 12 months) follow-up, LI interventions were superior to high intensity interventions at reducing eating disorder psychopathology (*n* = 4; *g* = − 0.20; 95% CI [− 0.40, − 0.01], *p* = 0.04). No indication for publication bias was found (*t* = 0.56, *p* = 0.60).

In relation to DSM severity specifier outcomes, there was a small but significant effect in favour of LI psychological interventions when compared to high intensity therapies (*g* = − 0.15; 95% CI [− 0.31, 0.00], *p* < 0.05; NNT = 11.11). There was no significant difference between low and high intensity interventions at short-term (*n* = 4; *g* = − 0.11; 95% CI [− 0.32, 0.10], *p* = 0.30) or long-term (≥ 12 months) follow-up (*n* = 3; *g* = − 0.12; 95% CI [− 0.32, 0.08], *p* = 0.22). There was no indication for publication bias (*t* = 0.84, *p* = 0.44).

There was an overall effect in favour of high intensity therapies compared with LI interventions on achieving remission and recovery (RR = 0.70; 95% CI [0.56, 0.87], *p* < 0.01). This means that provision of high intensity therapies increased the chances of remission and/or recovery by around 30%. At short-term follow-up, high and low intensity interventions were comparable in achieving remission and recovery (*n* = 4; RR = 0.68; 95% CI [0.64, 1.01], *p* = 0.07). There was no indication for publication bias (*t* = 0.67, *p* = 0.55).

##### Subgroup and moderator analyses

Meta-regression analyses showed no significant effect of total RoB score on effect size on any of the primary outcomes, and no significant association between age and effect size. There was no significant difference in effect across types of eating disorder, treatment modality, intervention format, mode of delivery, type of guidance or qualification of guide. All interventions included some form of guidance so it was not possible to compare guided and unguided interventions for these comparisons.

##### Summary statement

Compared to high intensity psychological interventions, low intensity psychological interventions appear to be equally efficacious at reducing eating disorder psychopathology, more effective on DSM severity specifier-related outcomes, and less likely to achieve remission and/or recovery.

#### Low intensity psychological interventions vs non-eating disorder-specific psychological interventions.

Effect size data for the seven studies comparing eating disorder-specific LI interventions against a non-eating disorder specific psychological intervention can be found in Table 4 (8 comparisons). Forest plots of effect sizes on each primary outcome for studies comparing against non-eating disorder specific interventions are presented in Additional file 5.1. Effect size data for each subgroup analyses are displayed in Additional file 5.2. See Additional file 5.3 for funnel plots examining publication bias.

In relation to eating disorder psychopathology, the pooled effect sizes were significantly greater for LI psychological interventions compared to non-eating disorder-specific interventions (*g* = − 0.35; 95% CI [− 0.49, − 0.22], *p* < 0.01; NNT = 5.10). These differences were no longer significant at short-term follow-up (*n* = 3; *g* = − 0.31; 95% CI [− 0.66, − 0.04], *p* = 0.08). No indication for publication bias was found (*t* = 0.42, *p* = 0.70).

Results also showed that LI psychological interventions had a small but significant effect on DSM severity specifier-related outcomes compared to non-eating disorder specific interventions (*g* = − 0.22; 95% CI [− 0.34, − 0.09], *p* < 0.01; NNT = 8.06), but comparable at short-term follow-up (*n* = 3; *g* = − 0.15; 95% CI [− 0.39, 0.04], *p* = 0.12). Visual inspection of a funnel plot indicated that the pooled effect size of studies comparing LI interventions against non-eating disorder specific interventions may have been influenced by publication bias, however Egger’s test was not significant (*t* = 1.87, *p* = 0.13). Following adjustment for missing studies using Duval and Tweedie’s (2000) trim-and-fill procedure (3 imputed studies), Hedges *g* was − 0.16 (95% CI − 0.26, − 0.06; NNT = 11.11).

There was an overall effect in favour of LI psychological interventions compared to non-eating disorder specific interventions on achieving remission and/or recovery (RR = 1.47; 95% CI [1.13, 1.92], *p* < 0.01), with those who received a LI intervention having an increased chance of remission and/or recovery of 47%. This effect increased and remained significant at short-term follow-up (*n* = 4; RR = 1.93; 95% CI [1.48, 0.53], *p* < 0.01). There was no indication for publication bias (*t* = 0.50, *p* = 0.64).

##### Subgroup and moderator analyses

Meta-regression analyses showed no significant effect of total RoB score on effect size on any of the primary outcomes, and there was no significant association between age and effect size. Subgroup analyses found no potential moderating effect among any of the variables investigated. All interventions were self-led so it was not possible to explore the moderating effect of ‘mode of delivery’.

##### Summary statement

Eating disorder-specific LI psychological interventions were superior to non-eating disorder specific psychological interventions across all three primary outcomes (eating disorder psychopathology, DSM severity specifier-related outcomes and rates of remission and/or recovery), with small but statistically significant effects.

#### Low intensity psychological interventions versus waiting list controls

Meta-analyses were performed at the post-intervention timepoint only. It was not possible to conduct analyses at follow-up due to trials using a crossover design, nor was it possible to explore the moderating effect of ‘mode of delivery’ as all interventions were self-led. Meta-regression analyses found no significant association between age and effect size on any of the comparisons. Effect size data for the 17 studies comparing against a waiting list control condition can be found in Table 5 (22 comparisons). Forest plots of effect sizes on each primary outcome for studies comparing against waiting list controls are presented in Additional file 6.1. Effect size data for each subgroup analyses are displayed in Additional file 6.2, and funnel plots examining publication bias are in Additional file 6.3.

For eating disorder psychopathology, the pooled effect sizes were moderate, statistically significant, and in favour of the LI psychological intervention (*g* = − 0.68; 95% CI [− 0.90, − 0.46]; *p* < 0.01; NNT = 2.70). However, Cochran’s *Q*-test identified moderately high heterogeneity across these studies (*I*^*2*^ = 67; *Q* = 42,* p* < 0.01). Meta-regression analyses revealed that the total RoB score had a significant effect on effect size (*z* = − 2.28,* p* = 0.02); only two studies with a waiting list condition had a total RoB score of ≤ 4. When considering moderators, there was a significant effect of ‘format of intervention’, with bibliotherapy (*n* = 8; *g* = − 0.93, 95% CI [− 1.28 to − 0.58]) superior to online (*n* = 5; *g* = − 52; 95% CI [− 0.69, − 0.35]) and CD-ROM interventions (*n* = 2; *g* = − 0.12; 95% CI [− 0.46, 0.21]). Subgroup analyses also revealed a moderating effect of ‘type of guidance’, with email guidance (*n* = 3; g = − 0.82; 95% CI [− 1.09, − 0.54]) more efficacious than online guidance (*n* = 2; *g* = − 0.39; 95% CI [− 0.61, − 0.16]). Visual inspection of a funnel plot indicated potential publication bias; however, Egger’s test was not significant (*t* = 1.97, *p* = 0.07) and Duval and Tweedie’s [47] trim-and-fill procedure resulted in no imputed studies.

Results showed a moderate effect in favour of LI psychological interventions on DSM severity specifier outcomes compared with waiting list (*g* = − 0.60; 95% CI [− 0.74, − 0.45], *p* < 0.01; NNT = 3.05). Meta-regression analyses revealed no significant effect of total RoB score on effect size, and no statistically significant differences among any of the subgroups investigated. A funnel plot indicated that the effect size may have been influenced by publication bias, although Egger’s test was not significant (*t* = 2.01, *p* = 0.07). Following adjustment for missing studies using Duval and Tweedie’s [47] trim-and-fill procedure (2 imputed studies), *g* was − 0.57 (95% CI [− 0.71, − 0.42]).

The effect of LI psychological interventions on achieving remission and/or recovery when compared to waiting list controls was RR = 3.01 (95% CI [1.93, 4.69], *p* < 0.01). This suggests that individuals who received a LI psychological intervention were 3× more likely to achieve remission and/or recovery than individuals waiting for treatment. However, a meta-regression analysis demonstrated that total RoB score was significantly associated with effect size (*z* = 1.94, *p* = 0.05); only one study in this comparison had a total RoB score of ≤ 4. Subgroup analyses found no significant differences between subgroups. A funnel plot indicated that the effect size was influenced by publication bias, which was confirmed by Egger's test (*t* = 3.02, *p* = 0.01). After adjusting for missing studies using Duval and Tweedie’s [47] trim-and-fill procedure (5 imputed studies), the RR reduced to 2.41 (95% CI [1.60, 3.62]).

#####  Summary statement

Compared to waitlist controls, low intensity psychological interventions demonstrated moderate effects on all three primary outcomes: eating disorder psychopathology, DSM severity specifier-related outcomes, and rates of remission and/or recovery.

## Discussion

This systematic review and meta-analysis aimed to systematically assess the evidence base for the use of LI psychological interventions for the treatment of feeding and eating disorders. The relative efficacy of LI psychological interventions was examined in comparison to high intensity psychological interventions, non-eating disorder specific psychological interventions and waiting list control conditions. Thirty-seven pooled comparisons using data from 30 studies were conducted.

Overall, findings suggest that LI psychological interventions can successfully treat eating disorder symptoms. Effect sizes varied as a function of the comparison condition. LI psychological interventions were superior to waiting list controls with moderate effects, demonstrated a small positive effect compared to non-eating disorder specific interventions, and were generally comparable to high intensity therapies at posttreatment. These findings are consistent with the pattern observed in prior meta-analyses of eating disorder treatments, which have also found strong effects for self-help compared to waiting list [33], and similar outcomes to therapist-delivered psychological therapies [83].

LI psychological interventions were consistently more efficacious than waiting list controls on all three primary outcomes, with an NNT of around three, indicating that one in every three patients will benefit from such an intervention. In these studies, there was evidence to suggest that self-help delivered via bibliotherapy may be favourable to computerised treatments. As the aim of this review was to examine the effectiveness of LI psychological interventions compared to other types of treatment and no treatment, RCTs comparing two types of the same intervention delivered through different formats were excluded from the current review, so this requires further investigation. In their RCT, Wagner et al. [84] compared two types of CBT guided self-help for BN (bibliotherapy vs internet-based) and found that internet-based guided self-help was not superior to its bibliotherapy equivalent. Given the shift towards e-mental health interventions in recent years, it is essential that more RCTs comparing different types of self-help (e.g., online vs bibliotherapy) are conducted in order to prevent the promulgation of ineffective or even harmful interventions [85].

This meta-analysis showed that, perhaps unsurprisingly, LI interventions with an emphasis on eating disorders were more effective at treating eating disorder symptoms than non-eating disorder specific interventions. Notably, however, the size of the pooled effect was smaller than that for studies with a waiting list control condition, which suggests interventions without an eating disorder focus (e.g., self-esteem self-help) may have some therapeutic benefit for individuals with eating disorders [56]. LI psychological interventions were generally comparable to therapist-delivered, high intensity therapies, although individuals were more likely to achieve remission and/or recovery if they received a more intensive treatment. However, these results should be interpreted with caution because of the limited quantity and quality of RCTs from which these conclusions have been drawn. There is a need for well-conducted trials exploring the effects of LI psychological interventions, particularly in comparison to specialist therapist-delivered therapies.

A number of reviews across mental health disorders have found guided self-help has greater adherence and effectiveness compared to self-help without guidance [86–88]. However, the subgroup analyses in this review revealed no significant differences in the effectiveness of LI psychological interventions with and without guidance. Trials comparing guided self-help to unguided self-help have had mixed results. Loeb et al. [89] found guided self-help to be superior in reducing the occurrence of binge eating, whereas Ghaderi et al. [90, 91] showed no significant differences between guided and unguided self-help in regards to eating disorder psychopathology. Although it was beyond the scope of the current review, direct comparisons of self-help with varying levels of guidance would be helpful.

Eating disorders are one of the most common problems in children and adolescents who access mental health services [92], and the number of young people needing treatment has reached record levels [93]. We know that early access to support is important for treatment outcomes [94], however only one RCT on an adolescent population fulfilled the inclusion criteria for this review. In their feasibility trial, Lock et al. [38] found adolescents with AN who underwent an online FBT guided self-help programme made clinical improvements in terms of weight gain and eating-related cognitions. There is also some evidence to suggest that guided self-help can be effective for adolescents with BN. Schmidt et al. [95] compared CBT guided self-care to family therapy in a sample of adolescents with BN and related disorders. The results indicated that CBT guided self-care offered a more rapid reduction of bingeing, as well as being regarded more acceptable and less expensive to administer. The amount of guidance in the guided self-care condition exceeded the definition of a LI psychological intervention of ≤ 6 h of therapist contact time [21], hence this study was not included in this review. Nevertheless, these findings suggest children and adolescents with eating disorders may well benefit from LI psychological interventions. More interventions which address the specific developmental needs of young people need to be developed, and then studied in large RCTs, before clinicians consider adopting this approach [34]. Future research should explore whether these interventions are more effective when targeted towards parents and carers (as in Lock et al. [38]), or toward the young person themselves (as in Schmidt et al. [95]).

This review highlights various other gaps in our knowledge about the effectiveness of LI psychological interventions for the treatment of feeding and eating disorders. Most of the LI psychological interventions studied in this meta-analysis were based on CBT principles, and while we attempted to investigate the potential moderating effects of treatment modality, these analyses were insufficiently powered to detect effects. As such, the empirical standing of other types of LI psychological interventions, such as DBT and FBT, is still unknown. Similarly, the majority of the studies in this review either recruited participants with eating disorders characterised by recurrent binge eating, or used the Overcoming Binge Eating [96] manual in their intervention. Very few studies focused on AN and atypical eating disorders (OSFED, formerly EDNOS), despite guided self-help being recommended for the latter [24]. The lack of studies on AN might be justified by cautiousness and concerns regarding the use of LI psychological interventions with individuals at risk of medical complications [18]. However, the recent findings from Lock et al. [38] RCT on adolescents with AN suggest that these interventions may be effective and acceptable for this population. No studies included participants with ARFID, pica or rumination disorder. Further research investigating the use of LI psychological interventions for the range of eating disorders currently under-represented in the literature is necessary.

### Limitations

Limitations to this meta-analysis must be considered. Firstly, the definition of a ‘low intensity’ psychological intervention (i.e., ≤ 6 h of therapist contact time; [21]) has the advantage that it is a published definition with the specific goal of facilitating meaningful synthesis across research studies, but also meant that some relevant papers were excluded from our analyses [95]. Secondly, the definitions of remission and/or recovery varied across studies but were aggregated in the analyses, which may have inadvertently affected the results. Experts within the field of eating disorders should look towards developing a common metric for remission and/or recovery, as has been done with other disorders (e.g., [97]). Moreover, the number of comparisons in many of the meta-analyses and subgroup analyses were low, and therefore possibly underpowered to make meaningful conclusions. In addition, the methodological quality of the studies in this meta-analysis was poor. Based on the criteria outlined in the Cochrane risk of bias tool [39], all studies were considered to be at high RoB. The most common problem, aside from a lack of blinding of participants to treatment condition which is common in psychological treatment studies, was a bias through missing outcome data. The possibility of publication bias is another limitation. Publication bias is a substantial problem for the credibility of meta-analytic results, as it yields overestimated effects and may suggest the presence of non-existent effects [98]. Although attempts were made to limit publication bias through grey literature searches and visual inspections of funnel plots [99], some unpublished trials could have been missed which may have inflated effect size estimates. Furthermore, the trim-and-fill method has been criticised for having a high false positive rate which needs to be considered when interpreting the findings [100]. Taking into account all of these limitations (i.e., low number of studies for many of the comparisons, high risk of bias and potential publication bias), the need for caution when interpreting the findings from this meta-analysis must be emphasised. More fully powered trials which address these limitations are warranted.

### Implications

Notwithstanding these shortcomings, these results have clear implications related to the use of LI psychological interventions for the treatment of eating disorders. In line with NICE recommendations for the treatment of adults with BED, BN and related disorders [24], our findings suggest LI CBT interventions seem to be an appropriate first step in a stepped care model of treatment delivery for adults with binge-eating related disorders. Given the similar effects to high intensity therapies, LI CBT interventions may also be a promising alternative to specialist treatment. Considering their relatively low costs and ease of accessibility, LI interventions have the potential to give people timely access to treatment for their eating disorder at a time when this is so desperately needed [9]. It is, of course, essential that patients’ needs and preferences, and the availability of resources, are taken into account when making treatment decisions. Further investigation into for whom LI psychological interventions do and do not work is an important research priority, to ensure that people who require more intensive support are not delayed from receiving the treatment they need. The number of potential moderators examined in this review was limited to reduce the likelihood of a false-positive result, as outlined in the Cochrane Handbook for Systematic Reviews of Interventions [101]. However, future research should conduct exploratory analyses on different moderators of treatment outcome and satisfaction (e.g., eating disorder severity, comorbidities etc.) to improve precision in matching LI psychological interventions to the needs and preferences of each individual [102].

## Conclusion

While the preliminary evidence for the potential efficacy of alternative LI interventions (e.g., FBT, DBT) looks promising, more research is needed before practitioners should adopt these treatments. The value of LI psychological interventions for children and adolescents, and people with AN, is at present uncertain, and nothing is currently known about its effect as a treatment for ARFID, pica or rumination disorder. More studies are required to establish the effectiveness of LI psychological interventions for these patient groups. The quality of these RCTs was far from optimal and more work needs to be done to ensure that future trials meet higher standards and can therefore offer more robust conclusions.

## Supplementary Information

**Additional file 1**. PRISMA Checklist.

**Additional file 2**. Search Terms.

**Additional file 3**. Attrition rates.

**Additional file 4**. Low intensity psychological interventions vs High intensity psychological interventions.

**Additional file 5**. Low intensity psychological interventions vs Non-eating disorder specific psychological interventions.

**Additional file 6**. Low intensity psychological interventions vs Waiting list controls.

## Data Availability

Data sharing is not applicable to this article as no datasets were generated or analysed during the current study.

## Abbreviations

LI: Low intensity
DSM: Diagnostic and statistical manual
RR: Risk ratio
AN: Anorexia nervosa
BN: Bulimia nervosa
BED: Binge eating disorder
ARFID: Avoidant/restrictive food intake disorder
OSFED: Other specified feeding or eating disorder
NNT: Number needed to treat
