# Supplementary material for: Low intensity psychological interventions for the treatment of feeding and eating disorders: a systematic review and meta-analysis
Source: J Eat Disord. 2023 Apr 4;11:56. doi: 10.1186/s40337-023-00775-2 (PMC10072817; doi:10.1186/s40337-023-00775-2)
Supplement: Supplementary file 2 — Additional file 2. Search Terms. [file 40337_2023_775_MOESM2_ESM.docx]

**Additional File 2. Search terms**

**Feeding and eating disorder:**

eating disorder*, feeding disorder* anorexi*, bulimi*, bing*, other specified feeding or eating disorder, OSFED, EDNOS, unspecified feeding or eating disorder, UFED, avoidant restrictive food intake disorder, ARFID, pica, rumination disorder

**Low intensity:**

low intensity, audio*, book*, distance*, homework, information, instruct*, instant messaging, iCBT, internet*, web*, phone, mobile, e-mail*, email*, leaflet*, material*, multi-media, multimedia, online*, on-line, pamphlet*, program*, remote, tele*, tape*, workbook*, self help, self-help, self change, self-change, self care, self-care, self directed, self-directed, self manage, self-manage, minimal guidance, minimal contact, bibliotherapy*, manual*, computer*, www, cd-rom, cd, cdrom, DVD, floppy, video*, virtual*

**Intervention:**

1. therap*
2. interven*
3. treat*
4. psychol*

**Randomised controlled trial:**

1. randomi*ed controlled trial*
2. clinical trial*
3. random*
4. allocat*
5. trial*
6. groups
