# Supplementary material for: Low intensity psychological interventions for the treatment of feeding and eating disorders: a systematic review and meta-analysis
Source: J Eat Disord. 2023 Apr 4;11:56. doi: 10.1186/s40337-023-00775-2 (PMC10072817; doi:10.1186/s40337-023-00775-2)
Supplement: Supplementary file 3 — Additional file 3. Attrition rates. [file 40337_2023_775_MOESM3_ESM.docx]

| **Additional File 3. Attrition rates**  Table AF.3  *Study attrition rates* | |
| --- | --- |
| Study | Main Findings |
| Bailer et al., 2004 | 10 out of 40 (25%) participants given the self-help manual did not complete the treatment phase. 15 out of 41 (36.6%) of those who received group CBT did not complete the treatment phase. The overall drop-out rate during the treatment phase was 30.8% (*n* = 25). The drop-out rate did not differ significantly between the two groups. Drop-outs did not differ significantly from completers on clinical or demographic variables at baseline. |
| Banasiak et al., 2005 | 18 out of 54 (33.3%) dropped out of the guided self-help intervention and 16 out of 55 (29.1%) dropped out of the delayed treatment control group. The overall attrition rate during the intervention phase was 31.2% (*n* = 34). There was no statistically significant difference between the two groups’ attrition rates. There were no significant differences between those who completed the trial and those who dropped out on demographic variables, clinical features or outcome variables at baseline. |
| Cachelin et al., 2019 | A total of 11 out of 40 (27.5%) participants did not complete the RCT. 6 out of 21 (28.6%) assigned to the guided self-help group did not complete the intervention. 5 out of the 19 (26.3%) participants in the waiting list condition did not complete the posttreatment assessment. The completer and non-completer groups were similar on all baseline demographic characteristics, help-seeking and outcome variables of interest. |
| Carrard et al., 2011 | 9 out of 37 (24.3%) participants allocated to the internet group and 4 out of 37 (10.8%) allocated to the delayed treatment group did not complete the posttreatment assessment. The overall attrition rate at posttreatment was 17.6% (13 out of 74). Dropouts had more concerns about shape and a higher drive for thinness compared to completers. Demographic characteristics and other outcome variables of both groups were similar at baseline. |
| Carter & Fairburn, 1998 | The overall attrition rate during the intervention phase was 12.5% (9 out of 72), 8 (33.3%) from the guided self-help group and 1 (4.2%) from the waiting list group. Five of the dropouts provided posttreatment data (1 from waiting list and 4 from guided self-help), so posttreatment data was available for 68 out of 72 (94.4%) participants. |
| Carter et al., 2003* | 20 out of 85 (23.5%) participants dropped out of the study and did not attend the posttreatment assessment: 5 (17.9%) were from the CBT self-help group, 7 (25%) from the nonspecific self-help group and 8 (27.6%) from the waiting list control group. There was no statistically significant difference between the three conditions in terms of rates of attrition. There were also no significant differences between the dropouts and completers in terms of baseline characteristics. |
| Carter et al., 2020 | 28 out of 74 (37.8%) did not complete the posttreatment questionnaires. At posttreatment, 29% in the DBT guided self-help group, 15% in the DBT unguided self-help group and 33% in the self-esteem unguided self-help group failed to provide data. There were no statistically significant differences between the three conditions in terms of attrition rates at posttreatment. Posttreatment completers reported lower baseline BSI-GSI scores than noncompleters, but these two groups did not differ on any other baseline variables. |
| de Zwaan et al., 2017 | Treatment attrition and study dropout during treatment were low, with an overall attrition rate of 9.6%. 13 out of 89 (14.6%) participants allocated to guided self-help intervention and 4 out of 89 (4.5%) allocated to CBT intervention did not provide posttreatment data. CBT was more successful in retaining patients in the trial than was guided self-help. |
| Duarte et al., 2017 | 6 out of 17 (35.3%) participants in the intervention group and 7 out of 16 (43.8%) in the waiting list control group did not complete the posttreatment assessments. The overall attrition rate at posttreatment was 39.4%. |
| Durand & King, 2003 | 12 out of 34 (35.3%) participants in the self-help group and 6 out of 34 (17.6%) participants in the specialist treatment group did not provide posttreatment data. The overall attrition rate at posttreatment was 26.5%. |
| Fitzsimmons-Craft et al., 2020 | Of the 385 participants randomised to the intervention condition, 158 (41%) did not complete the postintervention assessment. Of the 305 randomised to the control condition, 62 (20.3%) did not complete the postintervention assessment. The overall attrition rate at postintervention was 31.9%. |
| Green et al., 2018 | 17 out of the total 82 (20.1%) participants did not complete the postintervention assessment. |
| Grilo & Masheb, 2005 | Of the 90 participants, 70 (78%) completed treatment and 20 (22%) did not. Dropout rates were 13% for the CBT guided self-help group (*n* = 32 of 37), 34% (*n* = 13 of 38) for the BWL guided self-help group and 13% (*n* = 2 of 15) for the control group. The drop-out rate was significantly lower for CBT guided self-help compared to BWL guided self-help. |
| Grilo et al., 2013 | All 48 participants completed the RCT so the attrition rate was 0%. |
| Hildebrandt et al., 2020 | The total dropout rate was 32.8%, with dropout rates of 42.1% and 35.1% for the CBT guided self-help group and standard care groups, respectively. |
| Jenkins et al., 2021* | Across the two treatment conditions, only 50% of participants completed treatment. The attrition rate was 36.8% (*n* = 14 of 38) for the face-to-face group and 67.9% for the email group (*n* = 19 of 28). There was a greater dropout rate in the email supported self-help group compared to the face-to-face self-help group. |
| Kelly & Carter, 2015 | Out of the 41 participants in the study, 6 (14.6%) dropped out early or failed to provide data at the posttreatment assessment, 4 (26.6%) in the self-compassion intervention group, 1 (7.7%) in the behavioural strategies intervention group and 1 (7.7%) in the waiting list control group. |
| Ljotsson et al., 2007 | 11 of the 35 (31%) participants did not complete the full 12 weeks treatment program. Completers reported fewer subjective bulimic episodes than dropouts at the baseline assessment. All other baseline measures were equivalent across completers and dropouts. The overall attrition rate at posttreatment was 91.8% (*n* = 67 out of 73). Only 4 of the 37 (10.1%) participants in the treatment condition and 2 of the 36 (5.6%) participants in the waiting list condition failed to complete the post-treatment assessment. |
| Lock et al., 2021 | The overall attrition rate across treatment arms was 15% (*n* = 3). 2 out of 20 (10%) of participants in the FBT guided self-help condition dropped out of treatment and did not complete the end of treatment assessment. 2 of the 20 (10%) of participant in the high intensity FBT group dropped out of treatment, however only 1 of the 20 (5%) failed to provide posttreatment data. |
| Masson et al., 2013 | 9 of the 30 (30%) of the treatment group discontinued treatment and did not complete the posttreatment assessment. 3 of the 30 (10%) of the waiting list control group left the study prematurely. The overall attrition rate was 20%. |
| Palmer et al., 2002 | 30 out of the total 121 (24.8%) participants in the study dropped out of the study. Dropout rates across the four conditions were: 23.3% (*n* = 7) in the face-to-face guided self-help group, 25% (*n* = 7) in the telephone guided self-help group, 21.9% (*n* = 32) in the minimal guidance self-help group and 29% (*n* = 9) in the waiting list control group. |
| Peterson et al., 2020 | Of the 112 participants across both treatment arms, 23 (20.5%) did not complete treatment, including 16 (28.6%) in the CBT guided self-help group and 7 (12.5%) in the ICAT group. End of treatment assessments could not be obtained for 17 (30.3%) of CBT guided self-help group and 11 (19.6%) of the ICAT group. The overall attrition rate at posttreatment was 25%. ICAT was associated with a significantly higher treatment completion rates and lower drop out than CBT guided self-help. |
| Sánchez-Ortiz et al., 2011 | The overall attrition rate at posttreatment was 11.8%. 2 of the 38 (5.3%) in the CBT intervention group and 7 of the 38 (18.4%) in the delayed treatment control group did not complete posttreatment assessment measures. |
| Schmidt et al., 2008 | The overall attrition rate at posttreatment was 16.5%. 8 out of 49 (16.3%) in the CD-ROM group and 8 out of 48 (16.7%) in the waiting list control group failed to provide posttreatment data. |
| Shapiro et al., 2007 | Dropout rates within the intervention phase were 7 (31.5%) for the CD-ROM condition, 9 (40.9%) for the group CBT condition and 2 (9%) for the waiting list condition. There were significantly more dropouts in the CBT group condition compared to waiting list condition. The overall attrition rate at posttreatment was 27.3%. |
| Steele & Wade, 2008 | The attrition rate for participant in this trial was 25%, with a dropout rate of 4 (26.7%) for the CBT self-help group, 2 (11.8%) for the perfectionism self-help group and 4 (40%) in the placebo intervention group. |
| Strandskov et al., 2017 | Altogether, 19 participants dropped out of treatment during the treatment phase and another participant did not fill in the posttreatment assessment. The overall dropout rate was 21.7%. 15 (32.6%) of participants in the treatment group discontinued treatment prematurely and 4 (8.7%) participants in the waiting list control group dropped out. |
| Striegel-Moore et al., 2010 | Dropout was low, with only 11 out of 123 (8.9%) dropping out during the treatment phase. 7 out of 59 (11.9%) in the guided self-help group and 4 out of 64 (6.3%) in the usual care group did not complete the posttreatment assessment. |
| ter Huurne et al., 2015 | Within the web-based CBT group, 36 participants (33.3%) were considered treatment non-completers. Posttest assessments were not completed by 11 (10.2%) in the web-based CBT group and 2 (1.9%) in the waiting list group. The overall attrition rate at posttreatment was 5.4%. There was a higher study dropout in the web-based CBT group compared to waiting list. Participants who withdrew from the study more often lived alone and had less self-esteem at baseline than participants who completed the posttest. |
| Traviss et al., 2011 | The overall attrition rate from point of randomisation was 44.4%. 23 of 42 (54.8%) participants allocated to guided self-help failed to completed post-intervention measures. 13 of 39 (33.3%) participants in the waiting list control group did not complete post-intervention measures. |
| Treasure et al., 1994* | Of the randomised participants, 29 dropped out of treatment after starting: 14 were assigned to use the self-help manual, 7 were assigned to CBT and 8 were from the waiting list. It is not clear what percentage of participants randomised this was as the numbers reported do not include these participants (completer analyses). |
| Wilson et al., 2010 | At posttreatment, dropout rates were 30%, 7% and 28% for the CBT guided self-help group, IPT group and BWL group, respectively. The overall attrition rate for posttreatment assessments measures was 17.3% (*n* = 36 out of 208). IPT had a significantly lower attrition rate than both CBT guided self-help and BWL. |
| Wyssen et al., 2021 | 27% of all participants dropped out during the active treatment phase. Out of 24 participants who entered the online program, 8 (33.3%) dropped out during the active treatment phase. Dropout rates did not differ among the three groups. |
| *Note.* BWL = Behavioural Weight Loss; CBT = Cognitive Behavioural Therapy; DBT = Dialectical Behaviour Therapy; FBT = Family-Based Treatment; ICAT = Integrative Cognitive-Affective Therapy; IPT = Interpersonal Psychotherapy. | |
