# Supplementary material for: Low intensity psychological interventions for the treatment of feeding and eating disorders: a systematic review and meta-analysis
Source: J Eat Disord. 2023 Apr 4;11:56. doi: 10.1186/s40337-023-00775-2 (PMC10072817; doi:10.1186/s40337-023-00775-2)
Supplement: Supplementary file 4 — Additional file 4. Low intensity psychological interventions vs High intensity psychological interventions. [file 40337_2023_775_MOESM4_ESM.docx]

**Additional File 4. Low intensity psychological interventions vs High intensity psychological interventions**

1. Forest plots of effect sizes on each primary outcome for studies comparing against a high intensity psychological intervention

- [Eating disorder psychopathology](#Psychopathology)
- [DSM severity specifier-related outcomes](#Severity)
- [Remission and/or recovery rates](#Remission)

1. [Meta-analysis results](#Results) for studies comparing a low intensity psychological intervention against a high intensity psychological intervention on all three primary outcomes
2. [Funnel plots](#Funnel) with imputed studies for studies comparing a low intensity psychological intervention against a high intensity psychological intervention

| **Study name** | | **Hedge’s g** | **Lower limit** | **Upper limit** | ***p*-Value** | **Hedges’ g and 95% CI** | | | | | |
| --- | --- | --- | --- | --- | --- | --- | --- | --- | --- | --- | --- |
| Bailer 2004 | | -0.69 | -1.14 | -0.25 | 0.00 | 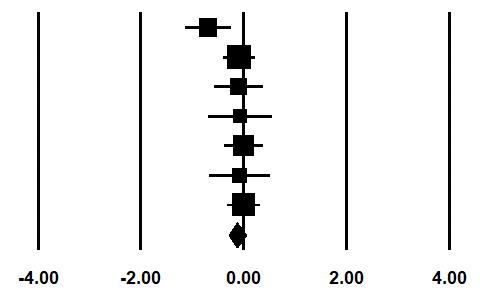 | | | | | |
| de Zwaan 2017 | | -0.09 | -0.39 | 0.21 | 0.55 |  |  |  |  |  |  |
| Durand 2003 | | -0.10 | -0.57 | 0.37 | 0.69 |  |  |  |  |  |  |
| Lock 2021 | | -0.07 | -0.68 | 0.53 | 0.81 |  |  |  |  |  |  |
| Peterson 2020 | | -0.01 | -0.37 | 0.36 | 0.98 |  |  |  |  |  |  |
| Shapiro 2007 | | -0.08 | -0.66 | 0.50 | 0.78 |  |  |  |  |  |  |
| Wilson 2010 | | 0.00 | -0.33 | 0.33 | 1.00 |  |  |  |  |  |  |
| **Vs. High Intensity Intervention Overall** | | **-0.13** | **-0.30** | **0.04** | **0.13** |  |  |  |  |  |  |
|  |  | |  |  |  | -4 | -2 | 0 | | 2 | 4 |
|  |  | |  |  |  | Favours low intensity intervention | | | Favours high intensity intervention | | |
| *Note.* Negative values favour low intensity psychological intervention. | | | | | | | | | | | |

**Figure AF4.1.1** *Forest plot of controlled between-group effect sizes for comparisons between low intensity psychological interventions and high intensity psychological interventions on eating disorder psychopathology*

**Figure AF4.1.2** *Forest plot of controlled between-group effect sizes for comparisons between low intensity psychological interventions and high intensity psychological interventions on DSM severity specifier-related outcomes*

| **Study name** | | **Hedge's g** | **Lower limit** | **Upper limit** | ***p*-Value** | **Hedges’ g and 95% CI** | | | | | |
| --- | --- | --- | --- | --- | --- | --- | --- | --- | --- | --- | --- |
| Bailer 2004 | | -0.19 | -0.63 | 0.24 | 0.38 | 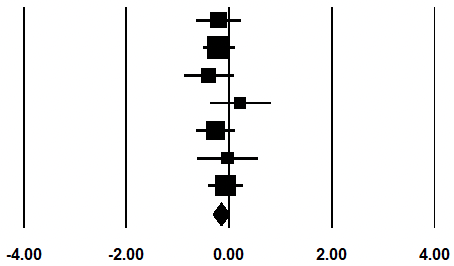 | | | | | |
| de Zwaan 2017 | | -0.19 | -0.49 | 0.11 | 0.21 |  |  |  |  |  |  |
| Durand 2003 | | -0.38 | -0.86 | 0.09 | 0.12 |  |  |  |  |  |  |
| Lock 2021 | | 0.23 | -0.38 | 0.84 | 0.46 |  |  |  |  |  |  |
| Peterson 2020 | | -0.25 | -0.62 | 0.11 | 0.18 |  |  |  |  |  |  |
| Shapiro 2007 | | -0.02 | -0.60 | 0.56 | 0.96 |  |  |  |  |  |  |
| Wilson 2010 | | -0.06 | -0.39 | 0.27 | 0.74 |  |  |  |  |  |  |
| **Vs. High Intensity Intervention Overall** | | **-0.15** | **-0.31** | **0.00** | **<0.05** |  |  |  |  |  |  |
|  |  | |  |  |  | -4 | -2 | 0 | | 2 | 4 |
|  |  | |  |  |  | Favours low intensity intervention | | | Favours high intensity intervention | | |
| *Note.* Negative values favour low intensity psychological intervention. | | | | | | | | | | | |

| **Study name** | | **Risk ratio** | **Lower limit** | **Upper limit** | ***p*-Value** | **Risk ratio and 95% CI** | | | | | |
| --- | --- | --- | --- | --- | --- | --- | --- | --- | --- | --- | --- |
| Bailer 2004 | | 0.92 | 0.32 | 2.64 | 0.87 | 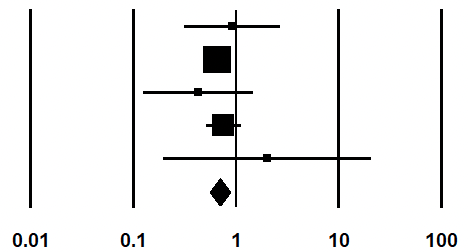 | | | | | |
| de Zwaan 2017 | | 0.66 | 0.49 | 0.88 | 0.01 |  |  |  |  |  |  |
| Lock 2021 | | 0.43 | 0.13 | 1.46 | 0.18 |  |  |  |  |  |  |
| Peterson 2020 | | 0.75 | 0.51 | 1.09 | 0.14 |  |  |  |  |  |  |
| Shapiro 2007 | | 2.00 | 0.20 | 20.49 | 0.56 |  |  |  |  |  |  |
| **Vs. High Intensity Intervention Overall** | | **0.70** | **0.56** | **0.87** | **<0.01** |  |  |  |  |  |  |
|  |  | |  |  |  | 0.01 | 0.1 | 1 | | 10 | 100 |
|  |  | |  |  |  | Favours high intensity intervention | | | Favours low intensity intervention | | |
| *Note.* Values greater than 1 favour low intensity psychological intervention. | | | | | | | | | | | |

**Figure AF4.1.3**

*Forest plot of controlled between-group effect sizes for comparisons between low intensity psychological interventions and high intensity psychological interventions on rates of remission and recovery*

**Table AF4.2** *Meta-analysis results for studies comparing a low intensity psychological intervention against a high intensity psychological intervention on all three primary outcomes*

|  | Ncomp | ES | 95%CI | *Z* | *I^2^* | *p* | | NNT | | *Q* (*p*) | |
| --- | --- | --- | --- | --- | --- | --- | --- | --- | --- | --- | --- |
| Eating disorder psychopathology (*g*) | 7 | -0.13 | -0.30 to 0.04 | -1.51 | 17.83 | .13 | 13.51 | | 7.30 (0.29) | |  |
| *Study characteristics* |  |  |  |  |  |  |  | |  | |  |
| Type of eating disorder |  |  |  |  |  |  |  | |  | |  |
| AN | 1 | -0.07 | -0.68 to 0.53 | -0.24 | <.001 | .52 | 25.00 | | <.01 (>.99) | |  |
| BED | 4 | -0.04 | -0.22 to 0.14 | -0.46 | <.001 |  | 45.45 | | 0.22 (0.97) | |  |
| BN | 2 | -0.40 | -0.98 to 0.18 | -1.34 | 69.11 |  | 4.50 | | 3.24 (0.07) | |  |
| Treatment modality |  |  |  |  |  |  |  | |  | |  |
| CBT | 6 | -0.14 | -0.34 to 0.05 | -1.42 | 31.23 | .84 | 12.82 | | 7.27 (0.20) | |  |
| FBT | 1 | -0.07 | -0.68 to 0.53 | -0.24 | <.001 |  | 25.00 | | <.01 (>.99) | |  |
| Format of intervention |  |  |  |  |  |  |  | |  | |  |
| Bibliotherapy | 4 | -0.18 | -0.49 to 0.13 | -1.15 | 57.94 | .90 | 9.80 | | 7.13 (0.07) | |  |
| CD-ROM | 1 | -0.08 | -0.66 to 0.50 | -0.28 | <.001 |  | 21.74 | | <.01 (>.99) | |  |
| Online | 2 | -0.09 | -0.36 to 0.18 | 0.53 | <.001 |  | 20.00 | | <.01 (0.71) | |  |
| Mode of delivery |  |  |  |  |  |  |  | |  | |  |
| Parent-led | 1 | -0.07 | -0.68 to 0.53 | -0.24 | <.001 |  | 25.00 | | <.01 (>.99) | |  |
| Self-led | 6 | -0.14 | -0.34 to 0.05 | -1.42 | 31.23 | .84 | 12.82 | | 7.27 (0.20) | |  |
| Type of guidance |  |  |  |  |  |  |  | |  | |  |
| Email | 1 | -0.09 | -0.39 to 0.21 | -0.59 | <.001 | .07 | 20.00 | | <.01 (>.99) | |  |
| Face-to-face | 1 | -0.69 | -1.14 to -0.25 | -3.05 | <.001 |  | 2.67 | | <.01 (>.99) | |  |
| Telephone | 1 | -0.08 | -0.66 to 0.50 | -0.28 | <.001 |  | 21.74 | | <.01 (>.99) | |  |
| Unknown | 4 | -0.03 | -0.23 to 0.18 | -0.27 | <.001 |  | 62.50 | | 0.15 (0.99) | |  |
| Qualification of guide |  |  |  |  |  |  |  | |  | |  |
| Eating disorder/CBT (or equivalent) specialist | 2 | -0.09 | -0.36 to 0.18 | -0.64 | <.001 | 0.69 | 20.00 | | <.01 (0.96) | |  |
| Non-specialist | 5 | -0.16 | -0.42 to 0.09 | -1.25 | 44.30 |  | 11.11 | | 7.18 (0.12) | |  |
|  |  |  |  |  |  |  |  | |  | |  |
| DSM severity specifier (*g*) | 7 | -0.15 | -0.31 to 0.00 | -1.99 | <.001 | <.05* | 11.11 | | 3.35 (0.76) | |  |
| *Study characteristics* |  |  |  |  |  |  |  | |  | |  |
| Type of eating disorder |  |  |  |  |  |  |  | |  | |  |
| AN | 1 | 0.23 | -0.38 to 0.84 | 0.74 | <.001 | .35 | 7.69 | | <.01 (>.99) | |  |
| BED | 4 | -0.15 | -0.33 to 0.03 | -1.62 | <.001 |  | 11.90 | | 0.90 (0.83) | |  |
| BN | 2 | -0.28 | -0.60 to 0.04 | -1.71 | <.001 |  | 4.50 | | 0.33 (0.57) | |  |
| Treatment modality |  |  |  |  |  |  |  | |  | |  |
| CBT | 6 | -0.18 | -0.34 to -0.02 | -2.25 | <.001 | .20 | 9.80 | | 1.71 (0.89) | |  |
| FBT | 1 | 0.23 | -0.38 to 0.84 | 0.74 | <.001 |  | 7.69 | | <.01 (>.99) | |  |
| Format of intervention |  |  |  |  |  |  |  | |  | |  |
| Bibliotherapy | 4 | -0.19 | -0.39 to <.01 | -1.95 | <.001 | .75 | 9.43 | | 1.38 (0.71) | |  |
| CD-ROM | 1 | -0.02 | -0.60 to 0.56 | -0.06 | <.001 |  | 83.33 | | <.01 (>.99) | |  |
| Online | 2 | -0.07 | -0.45 to 0.31 | -0.35 | 33.06 |  | 25.00 | | 1.50 (0.22) | |  |
| Mode of delivery |  |  |  |  |  |  |  | |  | |  |
| Parent-led | 1 | 0.23 | -0.38 to 0.84 | 0.74 | <.001 |  | 7.69 | | <.01 (>.99) | |  |
| Self-led | 6 | -0.18 | -0.34 to -0.02 | -2.25 | <.001 | .20 | 9.80 | | 1.71 (0.89) | |  |
| Type of guidance |  |  |  |  |  |  |  | |  | |  |
| Email | 1 | -0.19 | -0.49 to 0.11 | -1.25 | <.001 | .96 | 9.43 | | <.01 (>.99) | |  |
| Face-to-face | 1 | -0.19 | -0.63 to 0.24 | -0.87 | <.001 |  | 9.43 | | <.01 (>.99) | |  |
| Telephone | 1 | -0.02 | -0.60 to 0.56 | -0.06 | <.001 |  | 83.33 | | <.01 (>.99) | |  |
| Unknown | 4 | -0.15 | -0.35 to 0.06 | -1.38 | 1.15 |  | 11.90 | | 3.04 (0.39) | |  |
| Qualification of guide |  |  |  |  |  |  |  | |  | |  |
| Eating disorder/CBT (or equivalent) specialist | 2 | -0.07 | -0.45 to 0.31 | -0.35 | 33.06 | 0.61 | 25.00 | | 1.49 (0.22) | |  |
| Non-specialist | 5 | -0.18 | -0.36 to <.01 | -1.87 | <.001 |  | 9.80 | | 1.70 (0.79) | |  |
|  |  |  |  |  |  |  |  | |  | |  |
| Remission/recovery (RR) | 5 | 0.70 | 0.56 to 0.87 | -3.19 | <.001 | <.01** |  | | 1.94 (0.75) | |  |
| *Study characteristics* |  |  |  |  |  |  |  | |  | |  |
| Type of eating disorder |  |  |  |  |  |  |  | |  | |  |
| AN | 1 | 0.43 | 0.13 to 1.46 | -1.35 | <.001 | .65 |  | | <.01 (>.99) | |  |
| BED | 3 | 0.70 | 0.55 to 0.88 | -3.03 | <.001 |  |  | | 1.09 (0.58) | |  |
| BN | 1 | 0.92 | 0.32 to 2.64 | -0.16 | <.001 |  |  | | <.01 (>.99) | |  |
| Treatment modality |  |  |  |  |  |  |  | |  | |  |
| CBT | 4 | 0.71 | 0.56 to 0.89 | -2.99 | <.001 | .43 |  | | 1.33 (0.72) | |  |
| FBT | 1 | 0.43 | 0.13 to 1.46 | -1.35 | <.001 |  |  | | <.01 (>.99) | |  |
| Format of intervention |  |  |  |  |  |  |  | |  | |  |
| Bibliotherapy | 2 | -0.77 | 0.54 to 1.10 | -1.46 | <.001 | .50 |  | | 0.12 (0.73) | |  |
| CD-ROM | 1 | 2.00 | 0.20 to 20.49 | 0.58 | <.001 |  |  | | <.01 (>.99) | |  |
| Online | 2 | -0.64 | 0.48 to 0.86 | -3.02 | <.001 |  |  | | 0.44 (0.51) | |  |
| Mode of delivery |  |  |  |  |  |  |  | |  | |  |
| Parent-led | 1 | 0.43 | 0.13 to 1.46 | -1.35 | <.001 |  |  | | <.01 (>.99) | |  |
| Self-led | 4 | 0.71 | 0.56 to 0.89 | -2.99 | <.001 | .43 |  | | 1.33 (0.72) | |  |
| Type of guidance |  |  |  |  |  |  |  | |  | |  |
| Email | 1 | 0.66 | 0.49 to 0.88 | -2.77 | <.001 | .75 |  | | <.01 (>.99) | |  |
| Face-to-face | 1 | 0.92 | 0.32 to 2.64 | -0.16 | <.001 |  |  | | <.01 (>.99) | |  |
| Telephone | 1 | 2.00 | 0.20 to 20.49 | 0.58 | <.001 |  |  | | <.01 (>.99) | |  |
| Unknown | 2 | -0.71 | 0.50 to 1.03 | -1.82 | <.001 |  |  | | 0.72 (0.40) | |  |
| Qualification of guide |  |  |  |  |  |  |  | |  | |  |
| Eating disorder/CBT (or equivalent) specialist | 2 | 0.64 | 0.48 to 0.86 | -3.02 | <.001 | .39 |  | | 0.44 (0.51) | |  |
| Non-specialist | 3 | 0.78 | 0.55 to 1.12 | -1.35 | <.001 |  |  | | 0.76 (0.68) | |  |
| *Note.* For hedges’ *g,* negative values favour low intensity psychological intervention. For risk ratio, values > 1 favour low intensity psychological intervention.  AN = Anorexia Nervosa; BED = Binge Eating Disorder; BN = Bulimia Nervosa; CBT = Cognitive Behavioural Therapy; ES = Effect Size; FBT = Family-Based Treatment; Ncomp = Number of comparisons; NNT = Number Needed to Treat.  * *p* ≤ .05; ***p* ≤ .01. | | | | | | | | | | |  |

**Figure AF4.3** *Funnel plot with imputed studies for studies comparing low intensity psychological interventions against high intensity psychological interventions on (1) eating disorder psychopathology; (2) DSM severity specifier-related outcomes; and (3) rates of remission/recovery*


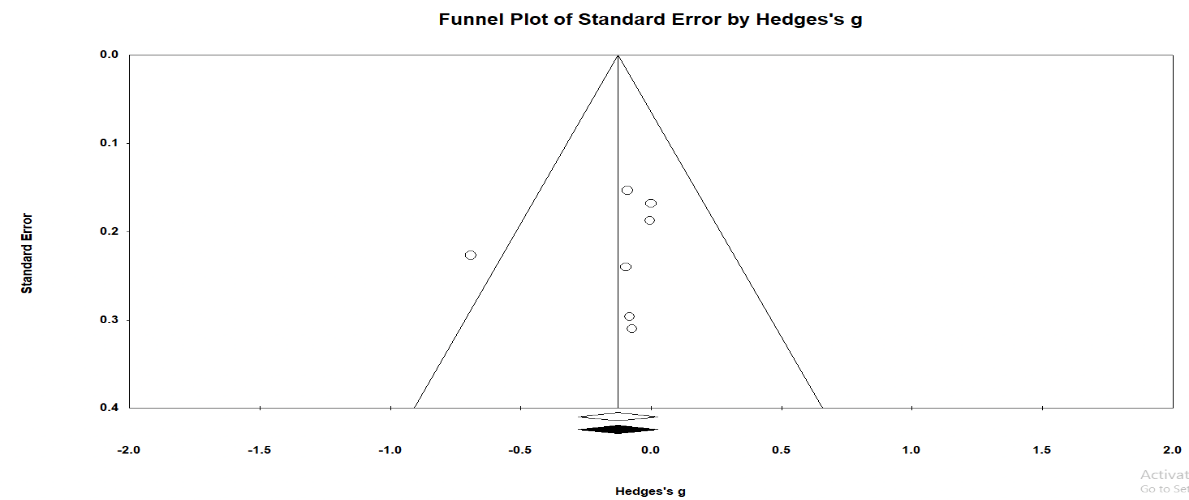

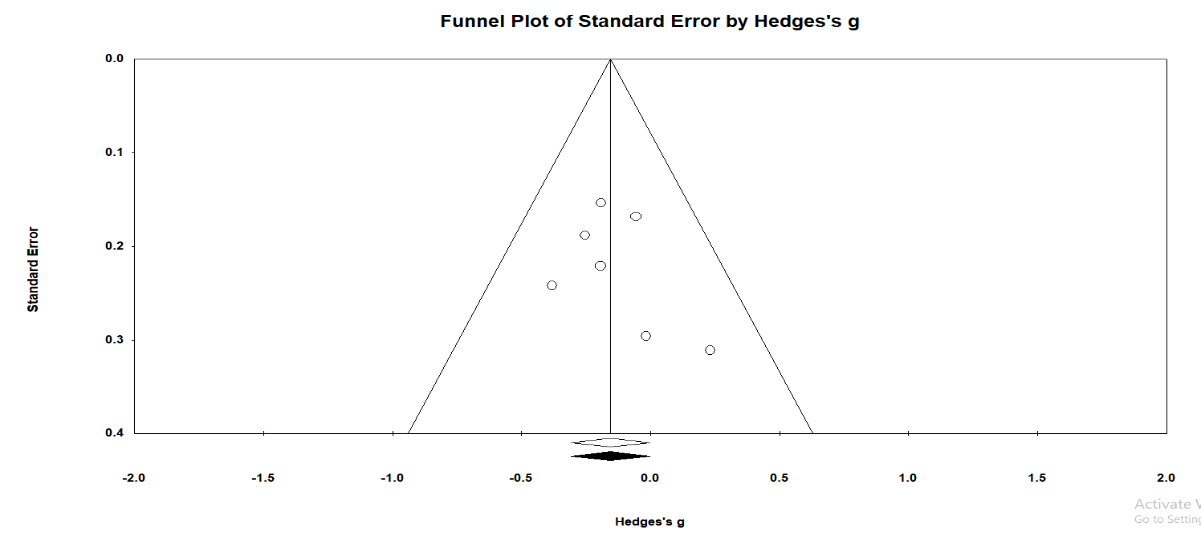

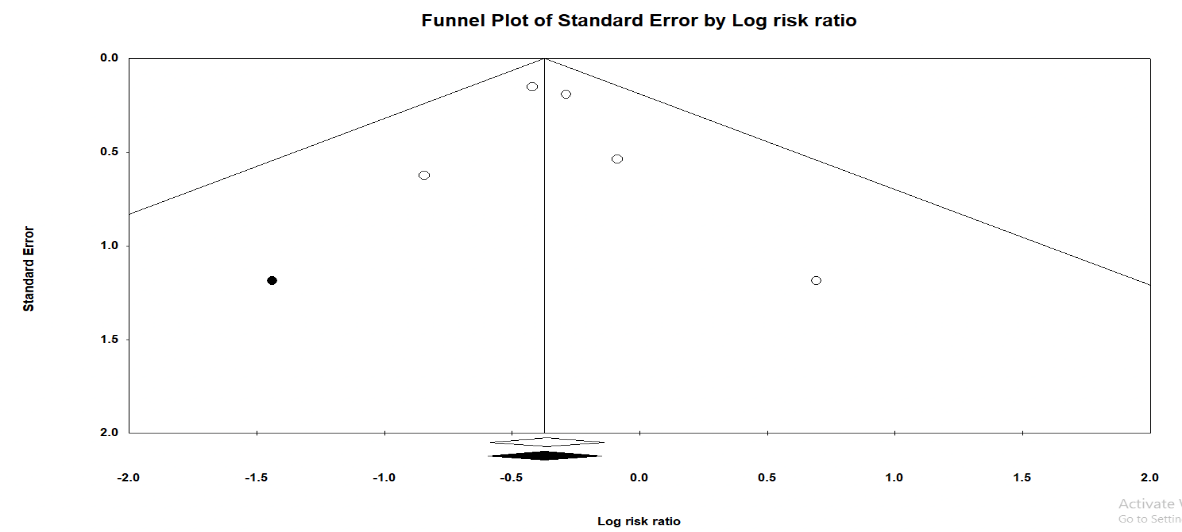


**(1)**

**(2)**

**(3)**
