# Supplementary material for: Low intensity psychological interventions for the treatment of feeding and eating disorders: a systematic review and meta-analysis
Source: J Eat Disord. 2023 Apr 4;11:56. doi: 10.1186/s40337-023-00775-2 (PMC10072817; doi:10.1186/s40337-023-00775-2)
Supplement: Supplementary file 5 — Additional file 5. Low intensity psychological interventions vs Non-eating disorder specific psychological interventions. [file 40337_2023_775_MOESM5_ESM.docx]

**Additional File 5. Eating disorder-specific low intensity psychological interventions vs Non-eating disorder specific psychological interventions**

1. Forest plots of effect sizes on each primary outcome for studies comparing against a non-eating disorder specific psychological intervention

- [Eating disorder psychopathology](#EatingDisorderPsychopathology)
- [DSM severity specifier-related outcomes](#DSMSeveritySpecifier)
- [Remission and/or recovery rates](#ForestPlot3)

1. [Meta-analysis results](#Results) for studies comparing an eating disorder-specific low intensity psychological intervention against a non-eating disorder specific psychological intervention on all three primary outcomes
2. [Funnel plots](#Funnel) with imputed studies for studies comparing an eating disorder-specific low intensity psychological intervention against a non-eating disorder specific psychological intervention

**Figure AF5.1.1** *Forest plot of controlled between-group effect sizes for comparisons between low intensity psychological interventions and non-eating disorder specific psychological interventions on eating disorder psychopathology*

| **Study name** | **Hedge's g** | **Lower limit** | **Upper limit** | ***p*-Value** | **Hedges’ g and 95% CI** | | | | | |
| --- | --- | --- | --- | --- | --- | --- | --- | --- | --- | --- |
| Carter 2020 (GSH) | -0.41 | -0.98 | 0.15 | 0.15 | 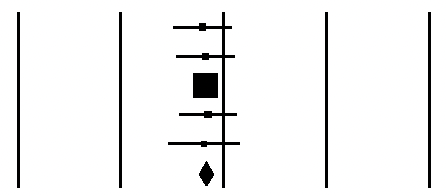 | | | | | |
| Carter 2020 (USH) | -0.35 | -0.92 | 0.22 | 0.22 |  |  |  |  |  |  |
| Fitzsimmons-Craft 2020 | -0.35 | -0.50 | -0.20 | 0.00 |  |  |  |  |  |  |
| Grilo 2013 | -0.30 | -0.86 | 0.26 | 0.29 |  |  |  |  |  |  |
| Steele 2008 | -0.38 | -1.07 | 0.32 | 0.29 |  |  |  |  |  |  |
| **Vs. Non-Eating Disorder Specific Intervention Overall** | **-0.35** | **-0.49** | **-0.22** | **<0.01** |  |  |  |  |  |  |
|  |  |  |  |  | -4 | -2 | 0 | | 2 | 4 |
|  |  |  |  |  | Favours low intensity intervention | | | Favours non-ED specific intervention | | |
| *Note.* Negative values favour low intensity psychological intervention. GSH = Guided self-help; USH = Unguided self-help. | | | | | | | | | | |

**Figure AF5.1.2** *Forest plot of controlled between-group effect sizes for comparisons between low intensity psychological interventions and non-eating disorder specific psychological interventions on DSM severity specifier-related outcomes*

| **Study name** | **Hedge's g** | **Lower limit** | **Upper limit** | ***p*-Value** | **Hedges’ g and 95% CI** | | | | | |
| --- | --- | --- | --- | --- | --- | --- | --- | --- | --- | --- |
| Carter 2020 (GSH) | -0.29 | -0.85 | 0.28 | 0.32 | 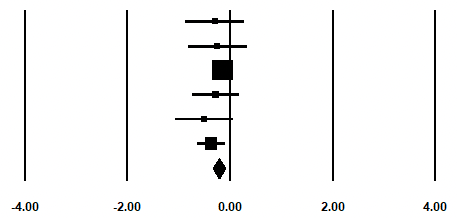 | | | | | |
| Carter 2020 (USH) | -0.24 | -0.81 | 0.32 | 0.40 |  |  |  |  |  |  |
| Fitzsimmons-Craft 2020 | -0.14 | -0.29 | 0.01 | 0.07 |  |  |  |  |  |  |
| Grilo 2005 | -0.27 | -0.72 | 0.18 | 0.23 |  |  |  |  |  |  |
| Grilo 2013 | -0.49 | -1.06 | 0.08 | 0.09 |  |  |  |  |  |  |
| Steele 2008 | -0.36 | -0.63 | -0.10 | 0.01 |  |  |  |  |  |  |
| **Vs. Non-Eating Disorder Specific Intervention Overall** | -0.22 | -0.34 | -0.10 | 0.00 |  |  |  |  |  |  |
|  |  |  |  |  | -4 | -2 | 0 | | 2 | 4 |
|  |  |  |  |  | Favours low intensity intervention | | | Favours non-ED specific intervention | | |
| *Note.* Negative values favour low intensity psychological intervention. GSH = Guided self-help; USH = Unguided self-help. | | | | | | | | | | |

| **Study name** | **Risk ratio** | **Lower limit** | **Upper limit** | ***p*-Value** | **Risk ratio and 95% CI** | | | | | |
| --- | --- | --- | --- | --- | --- | --- | --- | --- | --- | --- |
| Carter 2020 (GSH) | 1.17 | 0.60 | 2.29 | 0.64 | 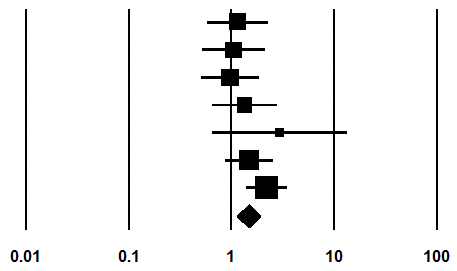 | | | | | |
| Carter 2020 (USH) | 1.06 | 0.53 | 2.13 | 0.86 |  |  |  |  |  |  |
| Fitzsimmons-Craft 2020 | 0.99 | 0.52 | 1.88 | 0.98 |  |  |  |  |  |  |
| Grilo 2005 | 1.37 | 0.66 | 2.86 | 0.40 |  |  |  |  |  |  |
| Grilo 2013 | 3.00 | 0.67 | 13.40 | 0.15 |  |  |  |  |  |  |
| Hildebrandt 2020 | 1.51 | 0.89 | 2.56 | 0.12 |  |  |  |  |  |  |
| Steele 2008 | 2.23 | 1.44 | 3.46 | 0.00 |  |  |  |  |  |  |
| **Vs. Non-Eating Disorder Specific Intervention Overall** | **1.47** | **1.13** | **1.91** | **0.00** |  |  |  |  |  |  |
|  |  |  |  |  | 0.01 | 0.1 | 1 | | 10 | 100 |
|  |  |  |  |  | Favours non-ED specific intervention | | | Favours low intensity intervention | | |
| *Note.* Values greater than 1 favour low intensity psychological intervention. GSH = Guided self-help; USH = Unguided self-help. | | | | | | | | | | |

**Figure AF5.1.3**

*Forest plot of controlled between-group effect sizes for comparisons between low intensity psychological interventions and non-eating disorder*

**Table AF5.2** *Meta-analysis results for studies comparing a low intensity psychological intervention against a non-eating disorder specific psychological intervention on all three primary outcomes*

|  | Ncomp | ES | 95%CI | *Z* | *I^2^* | *p* | | NNT | | *Q* (*p*) | |
| --- | --- | --- | --- | --- | --- | --- | --- | --- | --- | --- | --- |
| Eating disorder psychopathology (*g*) | 5 | -0.35 | -0.49 to -0.22 | -5.11 | <.001 | .<.01** | 5.10 | | 0.09 (>.99) | |  |
| *Study characteristics* |  |  |  |  |  |  |  | |  | |  |
| Type of eating disorder |  |  |  |  |  |  |  | |  | |  |
| BED | 3 | -0.36 | -0.68 to -0.03 | -2.13 | <.001 | >.99 | 5.00 | | 0.08 (0.96) | |  |
| BN | 1 | -0.38 | -1.07 to 0.32 | -1.07 | <.001 |  | 4.72 | | <.01 (>.99) | |  |
| Mixed | 1 | -0.35 | -0.50 to -0.20 | -4.52 | <.001 |  | 5.10 | | <.01 (>.99) | |  |
| Treatment modality |  |  |  |  |  |  |  | |  | |  |
| CBT | 3 | -0.35 | -0.49 to -0.20 | -4.76 | <.001 | .87 | 5.10 | | 0.03 (0.98) | |  |
| DBT | 2 | -0.38 | -0.78 to 0.02 | -1.87 | <.001 |  | 4.72 | | 0.02 (0.88) | |  |
| Format of intervention |  |  |  |  |  |  |  | |  | |  |
| Bibliotherapy | 4 | -0.36 | -0.65 to -0.06 | -2.38 | <.001 | .95 | 5.00 | | 0.08 (0.99) | |  |
| Online | 1 | -0.35 | -0.50 to -0.20 | -4.52 | <.001 |  | 5.10 | | <.01 (>.99) | |  |
| Provision of guidance |  |  |  |  |  |  |  | |  | |  |
| Guided | 3 | -0.35 | -0.50 to -0.21 | -4.85 | <.001 | .90 | 5.10 | | 0.05 (0.97) | |  |
| Unguided | 2 | -0.33 | -0.72 to 0.07 | -1.60 | <.001 |  | 5.43 | | 0.02 (0.90) | |  |
| Type of guidance |  |  |  |  |  |  |  | |  | |  |
| Online | 1 | -0.35 | -0.50 to -0.20 | -4.52 | <.001 | .97 | 5.10 | | <.01 (>.99) | |  |
| Unknown | 1 | -0.38 | -1.07 to 0.32 | -1.07 | <.001 |  | 4.72 | | <.01 (>.99) | |  |
| Video call | 1 | -0.41 | -0.98 to 0.15 | -1.43 | <.001 |  | 4.39 | | <.01 (>.99) | |  |
|  |  |  |  |  |  |  |  | |  | |  |
|  | | | | | | | | | | |  |
| DSM severity specifier (*g*) | 6 | -0.22 | -0.34 to -0.10 | -3.66 | <.001 | <.01** | 8.06 | | 3.20 (0.67) | |  |
| *Study characteristics* |  |  |  |  |  |  |  | |  | |  |
| Type of eating disorder |  |  |  |  |  |  |  | |  | |  |
| BED | 4 | -0.32 | -0.58 to -0.05 | -2.34 | <.001 | .60 | 5.56 | | 0.47 (0.93) | |  |
| Mixed | 2 | -0.23 | -0.44 to -0.01 | -2.07 | 52.29 |  | 7.69 | | 2.10 (0.15) | |  |
| Treatment modality |  |  |  |  |  |  |  | |  | |  |
| CBT | 4 | -0.22 | -0.35 to -0.09 | -3.33 | 4.35 | .84 | 8.06 | | 3.14 (0.37) | |  |
| DBT | 2 | -0.26 | -0.66 to 0.14 | -1.29 | <.001 |  | 6.85 | | <.01 (>.99) | |  |
| Format of intervention |  |  |  |  |  |  |  | |  | |  |
| Bibliotherapy | 5 | -0.34 | -0.53 to -0.15 | -3.57 | <.001 | .10 | 5.26 | | 0.53 (0.97) | |  |
| Online | 1 | -0.14 | -0.29 to 0.01 | -1.83 | <.001 |  | 12.83 | | <.01 (>.99) | |  |
| Provision of guidance |  |  |  |  |  |  |  | |  | |  |
| Guided | 4 | -0.21 | -0.33 to -0.08 | -3.29 | <.001 | .46 | 8.47 | | 2.29 (0.52) | |  |
| Unguided | 2 | -0.36 | -0.77 to 0.04 | -1.77 | <.001 |  | 5.00 | | 0.37 (0.54) | |  |
| Type of guidance |  |  |  |  |  |  |  | |  | |  |
| Face-to-face | 1 | -0.28 | -0.73 to 0.18 | -1.20 | <.001 | .51 | 6.41 | | <.01 (>.99) | |  |
| Online | 1 | -0.14 | -0.29 to 0.01 | -1.83 | <.001 |  | 12.82 | | <.01 (>.99) | |  |
| Telephone | 1 | -0.36 | -0.63 to -0.10 | -2.71 | <.001 |  | 5.00 | | <.01 (>.99) | |  |
| Video call | 1 | -0.29 | -0.85 to 0.28 | -0.99 | <.001 |  | 6.17 | | <.01 (>.99) | |  |
|  |  |  |  |  |  |  |  | |  | |  |
| Remission/recovery (RR) | 7 | 1.47 | 1.13 to 1.92 | 2.87 | 15.48 | <.01** |  | | 7.10 (0.31) | |  |
| *Study characteristics* |  |  |  |  |  |  |  | |  | |  |
| Type of eating disorder |  |  |  |  |  |  |  | |  | |  |
| BED | 4 | 1.27 | 0.86 to 1.87 | 1.19 | <.001 | .48 |  | | 1.61 (0.66) | |  |
| Mixed | 3 | 1.57 | 1.00 to 2.46 | 1.95 | 54.14 |  |  | | 4.36 (0.11) | |  |
| Treatment modality |  |  |  |  |  |  |  | |  | |  |
| CBT | 5 | 1.61 | 1.61 to 2.23 | 2.86 | 23.35 | .22 |  | | 5.22 (0.27) | |  |
| DBT | 2 | 1.12 | 0.69 to 1.81 | 0.46 | <.001 |  |  | | 0.04 (0.85) | |  |
| Format of intervention |  |  |  |  |  |  |  | |  | |  |
| Bibliotherapy | 6 | 1.59 | 1.22 to 2.07 | 3.45 | 4.63 | .18 |  | | 5.24 (0.39) | |  |
| Online | 1 | 0.99 | 0.52 to 1.88 | -0.03 | <.001 |  |  | | <.01 (>.99) | |  |
| Provision of guidance |  |  |  |  |  |  |  | |  | |  |
| Guided | 5 | 1.49 | 1.11 to 2.02 | 2.62 | 24.67 | .93 |  | | 5.31 (0.26) | |  |
| Unguided | 2 | 1.43 | 0.57 to 3.59 | 0.77 | 33.89 |  |  | | 1.51 (0.22) | |  |
| Type of guidance |  |  |  |  |  |  |  | |  | |  |
| Face-to-face | 1 | 1.37 | 0.66 to 2.86 | 0.84 | <.001 | .26 |  | | <.01 (>.99) | |  |
| Online | 1 | 0.99 | 0.52 to 1.88 | -0.03 | <.001 |  |  | | <.01 (>.99) | |  |
| Telephone | 1 | 1.51 | 0.89 to 2.57 | 1.54 | <.001 |  |  | | <.01 (>.99) | |  |
| Unknown | 1 | 2.23 | 1.44 to 3.46 | 3.59 | <.001 |  |  | | <.01 (>.99) | |  |
| Video call | 1 | 1.17 | 0.60 to 2.29 | 0.46 | <.001 |  |  | | <.01 (>.99) | |  |
| Qualification of guide |  |  |  |  |  |  |  | |  | |  |
| Eating disorder/CBT specialist (or equivalent) | 1 | 1.37 | 0.66 to 2.86 | 0.84 | <.001 | .12 |  | | <.01 (>.99) | |  |
| Mental health specialist | 1 | 2.23 | 1.44 to 3.46 | 3.59 | <.001 |  |  | | <.01 (>.99) | |  |
| Non-specialist | 3 | 1.25 | 0.88 to 1.77 | 1.24 | <.001 |  |  | | <.01 (>.99) | |  |
| *Note.* For hedges’ *g,* negative values favour eating disorder-specific low intensity psychological intervention. For risk ratio, values > 1 favours eating disorder-specific low intensity psychological intervention.  BED = Binge Eating Disorder; CBT = Cognitive Behavioural Therapy; DBT = Dialectical Behaviour Therapy; ES = Effect Size. Ncomp = Number of comparisons; NNT = Number Needed to Treat.  * *p* ≤ .05; ***p* ≤ .01. | | | | | | | | | | |  |

**Figure AF5.3**

*Funnel plot with imputed studies for studies comparing low intensity psychological interventions against non-eating disorder specific psychological interventions on (1) eating disorder psychopathology; (2) DSM severity specifier-related outcomes; and (3) rates of remission/recovery*


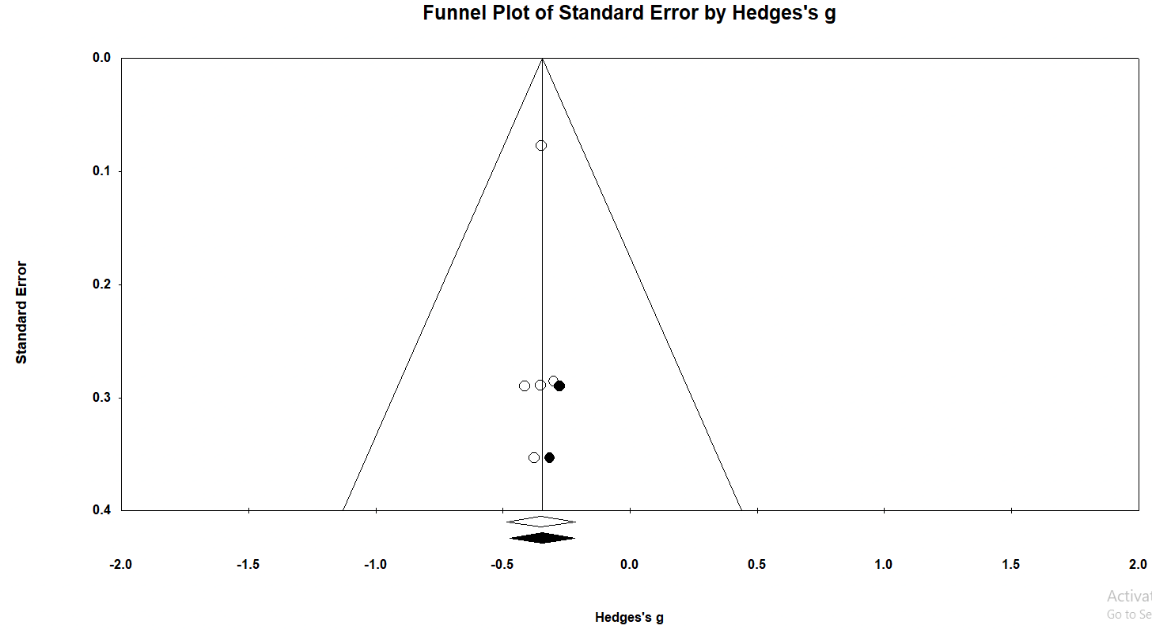


**(1)**


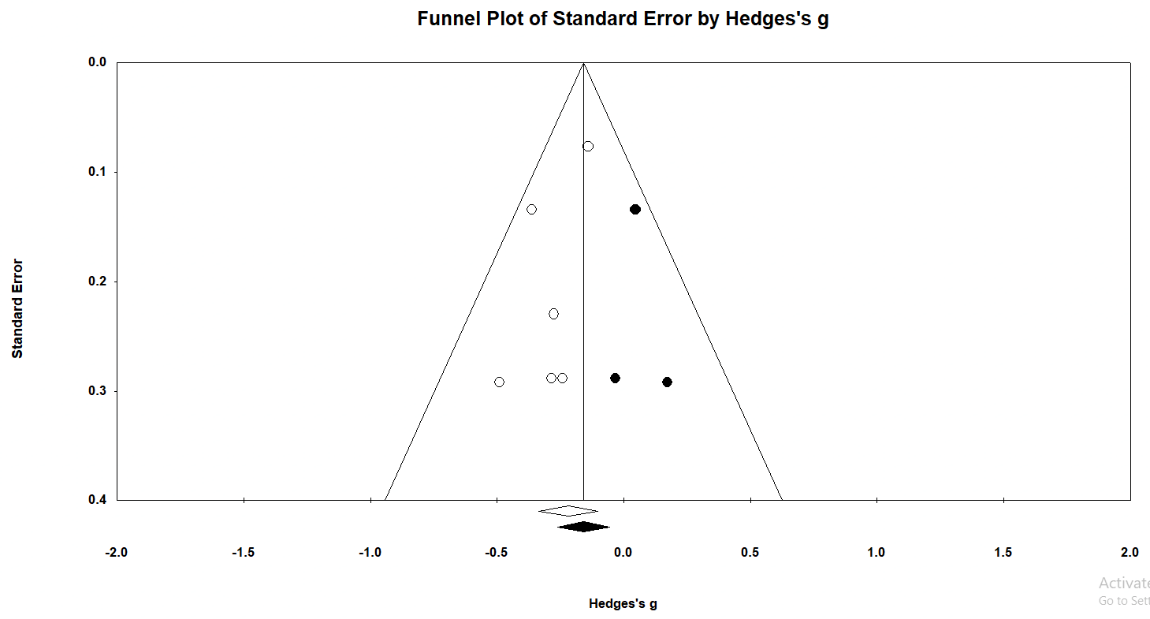


**(2)**


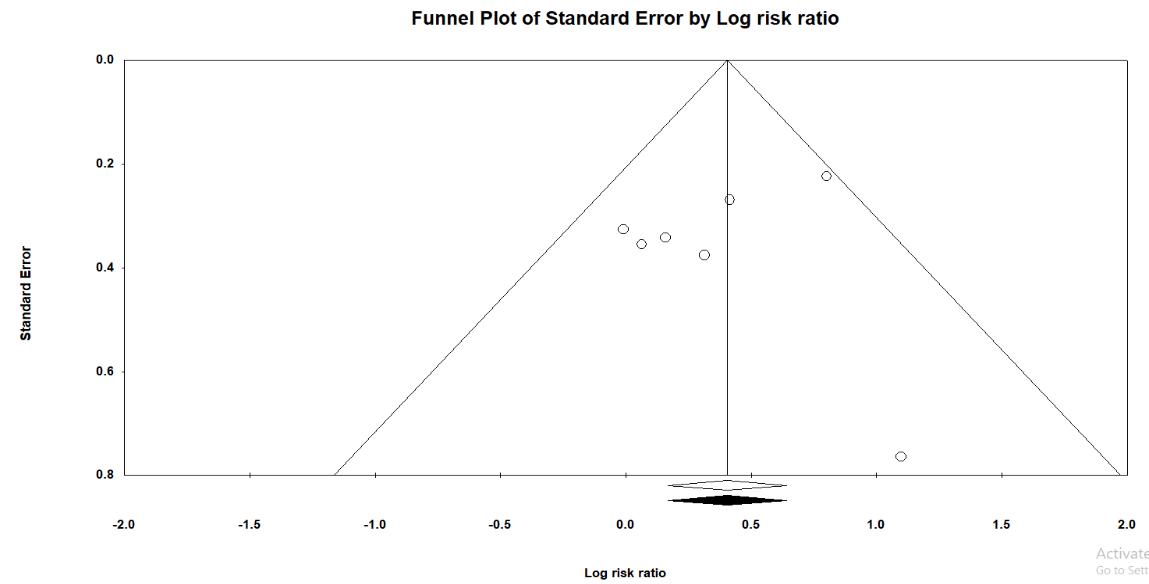


**(3)**
