# Supplementary material for: Low intensity psychological interventions for the treatment of feeding and eating disorders: a systematic review and meta-analysis
Source: J Eat Disord. 2023 Apr 4;11:56. doi: 10.1186/s40337-023-00775-2 (PMC10072817; doi:10.1186/s40337-023-00775-2)
Supplement: Supplementary file 6 — Additional file 6. Low intensity psychological interventions vs Waiting list controls. [file 40337_2023_775_MOESM6_ESM.docx]

**Additional File 6. Low intensity psychological interventions vs Waiting list control conditions**

1. Forest plots of effect sizes on each primary outcome for studies comparing against a waiting list control condition

- [Eating disorder psychopathology](#EatingDisorderPsychopathology)
- [DSM severity specifier-related outcomes](#DSMSeveritySpecifier)
- [Remission and/or recovery rates](#RemissionRecovery)

1. [Meta-analysis results](#Results) for studies comparing a low intensity psychological intervention against waiting list controls on all three primary outcomes
2. [Funnel plots](#Funnel) with imputed studies for studies comparing a low intensity psychological intervention against a waiting list control condition

| **Study name** | **Hedge's g** | **Lower limit** | **Upper limit** | ***p*-Value** | **Hedges’ g and 95% CI** | | | | | |
| --- | --- | --- | --- | --- | --- | --- | --- | --- | --- | --- |
| Banasiak 2005 | -0.81 | -1.20 | -0.43 | 0.00 | 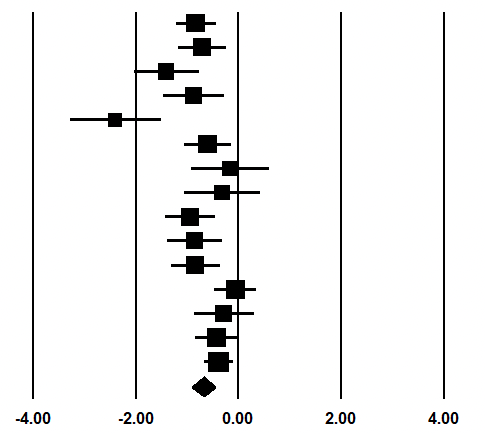 | | | | | |
| Carrard 2011 | -0.70 | -1.16 | -0.23 | 0.00 |  |  |  |  |  |  |
| Carter 1998 (GSH) | -1.39 | -2.01 | -0.77 | 0.00 |  |  |  |  |  |  |
| Carter 1998 (USH) | -0.86 | -1.44 | -0.28 | 0.00 |  |  |  |  |  |  |
| Duarte 2017 | -2.39 | -3.27 | -1.51 | 0.00 |  |  |  |  |  |  |
| Green 2018 | -0.59 | -1.03 | -0.15 | 0.01 |  |  |  |  |  |  |
| Kelly 2015 (Behavioural-SH) | -0.16 | -0.90 | 0.59 | 0.68 |  |  |  |  |  |  |
| Kelly 2015 (CFT-SH) | -0.30 | -1.03 | 0.42 | 0.41 |  |  |  |  |  |  |
| Ljotsson 2007 | -0.93 | -1.40 | -0.45 | 0.00 |  |  |  |  |  |  |
| Masson 2013 | -0.84 | -1.37 | -0.32 | 0.00 |  |  |  |  |  |  |
| Sánchez-Ortiz 2011 | -0.83 | -1.29 | -0.36 | 0.00 |  |  |  |  |  |  |
| Schmidt 2008 | -0.05 | -0.44 | 0.35 | 0.81 |  |  |  |  |  |  |
| Shapiro 2007 | -0.27 | -0.85 | 0.31 | 0.36 |  |  |  |  |  |  |
| Strandskov 2017 | -0.42 | -0.83 | -0.01 | 0.05 |  |  |  |  |  |  |
| ter Huurne 2015 | -0.37 | -0.64 | -0.10 | 0.01 |  |  |  |  |  |  |
| **vs. Waiting list Control Overall** | **-0.68** | **-0.89** | **-0.46** | **<0.01** |  |  |  |  |  |  |
|  |  |  |  |  | -4 | -2 | 0 | | 2 | 4 |
|  |  |  |  |  | Favours low intensity intervention | | | Favours waiting list control | | |
| *Note.* Negative values favour low intensity psychological intervention. Behavioural-SH = Behavioural guided self-help; CFT-GSH = Compassion-focused therapy-based self-help; GSH = Guided self-help; USH = Unguided self-help. | | | | | | | | | | |

**Figure AF6.1.1** *Forest plot of controlled between-group effect sizes for comparisons between low intensity psychological interventions and waiting list controls on eating disorder psychopathology*

| **Study name** | **Hedge's g** | **Lower limit** | **Upper limit** | ***p*-Value** | **Hedges’ g and 95% CI** | | | | | |
| --- | --- | --- | --- | --- | --- | --- | --- | --- | --- | --- |
| Banasiak 2005 | -0.58 | -0.96 | -0.20 | 0.00 | 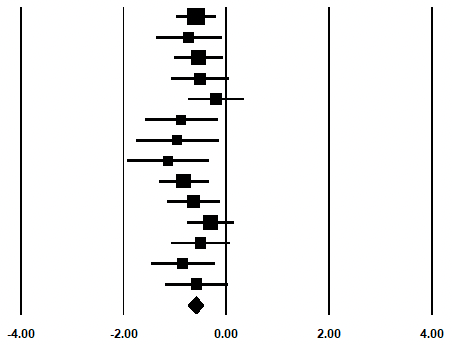 | | | | | |
| Cachelin 2019 | -0.72 | -1.34 | -0.09 | 0.03 |  |  |  |  |  |  |
| Carrard 2011 | -0.53 | -0.99 | -0.07 | 0.02 |  |  |  |  |  |  |
| Carter 1998 (GSH) | -0.50 | -1.07 | 0.06 | 0.08 |  |  |  |  |  |  |
| Carter 1998 (USH) | -0.19 | -0.75 | 0.37 | 0.51 |  |  |  |  |  |  |
| Duarte 2017 | -0.86 | -1.56 | -0.16 | 0.02 |  |  |  |  |  |  |
| Kelly 2015 (Behavioural-SH) | -0.94 | -1.73 | -0.15 | 0.02 |  |  |  |  |  |  |
| Kelly 2015 (CFT-SH) | -1.12 | -1.90 | -0.33 | 0.01 |  |  |  |  |  |  |
| Ljotsson 2007 | -0.82 | -1.29 | -0.35 | 0.00 |  |  |  |  |  |  |
| Masson 2013 | -0.62 | -1.14 | -0.11 | 0.02 |  |  |  |  |  |  |
| Sánchez-Ortiz 2011 | -0.29 | -0.73 | 0.16 | 0.21 |  |  |  |  |  |  |
| Shapiro 2007 | -0.49 | -1.08 | 0.10 | 0.10 |  |  |  |  |  |  |
| Wyssen 2021 | -0.84 | -1.45 | -0.23 | 0.01 |  |  |  |  |  |  |
| Wyssen 2021 | -0.57 | -1.17 | 0.03 | 0.06 |  |  |  |  |  |  |
| **vs. Waiting list Control Overall** | -0.60 | -0.74 | -0.45 | 0.00 |  |  |  |  |  |  |
|  |  |  |  |  | -4 | -2 | 0 | | 2 | 4 |
|  |  |  |  |  | Favours low intensity intervention | | | Favours waiting list control | | |
| *Note.* Negative values favour low intensity psychological intervention. Behavioural-SH = Behavioural guided self-help; CFT-GSH = Compassion-focused therapy-based self-help; GSH = Guided self-help; USH = Unguided self-help. | | | | | | | | | | |

**Figure AF6.1.2** *Forest plot of controlled between-group effect sizes for comparisons between low intensity psychological interventions and waiting list controls on DSM severity specifier-related outcomes*

| **Study name** | **Risk ratio** | **Lower limit** | **Upper limit** | ***p*-Value** | **Risk ratio and 95% CI** | | | | | |
| --- | --- | --- | --- | --- | --- | --- | --- | --- | --- | --- |
| Banasiak 2005 | 2.36 | 1.02 | 5.47 | 0.05 | 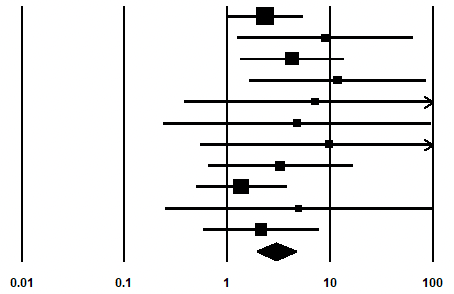 | | | | | |
| Cachelin 2019 | 9.05 | 1.27 | 64.21 | 0.03 |  |  |  |  |  |  |
| Carrard 2011 | 4.33 | 1.35 | 13.96 | 0.01 |  |  |  |  |  |  |
| Masson 2013 | 12.00 | 1.66 | 86.59 | 0.01 |  |  |  |  |  |  |
| Palmer 2002 (GSH-F) | 7.23 | 0.39 | 134.21 | 0.18 |  |  |  |  |  |  |
| Palmer 2002 (SH-MG) | 4.85 | 0.24 | 97.11 | 0.30 |  |  |  |  |  |  |
| Palmer 2002 (GSH-T) | 9.93 | 0.56 | 176.60 | 0.12 |  |  |  |  |  |  |
| Sánchez-Ortiz 2011 | 3.35 | 0.66 | 16.87 | 0.14 |  |  |  |  |  |  |
| Schmidt 2008 | 1.39 | 0.51 | 3.80 | 0.53 |  |  |  |  |  |  |
| Shapiro 2007 | 5.00 | 0.25 | 98.52 | 0.29 |  |  |  |  |  |  |
| Traviss 2011 | 2.17 | 0.60 | 7.80 | 0.24 |  |  |  |  |  |  |
| **vs. Waiting list Control Overall** | **3.01** | **1.93** | **4.69** | **0.00** |  |  |  |  |  |  |
|  |  |  |  |  | 0.01 | 0.1 | 1 | | 10 | 100 |
|  |  |  |  |  | Favours waiting list control | | | Favours low intensity intervention | | |
| *Note.* Values greater than 1 favour low intensity psychological intervention. GSH-F = GSH with face-to-face guidance; GSH-MG = Self-help with minimal guidance; GSH-T = GSH with telephone guidance. | | | | | | | | | | |

**Figure AF6.1.3** *Forest plot of controlled between-group effect sizes for comparisons between low intensity psychological interventions and waiting list controls on rates of remission and recovery*

**Table AF6.2** *Meta-analysis results for studies comparing a low intensity psychological intervention against waiting list controls on all three primary outcomes*

|  | Ncomp | ES | 95%CI | *Z* | *I^2^* | *p* | | NNT | | *Q* (*p*) | |
| --- | --- | --- | --- | --- | --- | --- | --- | --- | --- | --- | --- |
| Eating disorder psychopathology (*g*) | 15 | -0.68 | -0.90 to -0.46 | -6.05 | 66.57 | <.01** | 2.70 | | 41.88 (<.01) | |  |
| *Study characteristics* |  |  |  |  |  |  |  | |  | |  |
| Type of eating disorder |  |  |  |  |  |  |  | |  | |  |
| BED | 9 | -0.82 | -1.16 to -0.49 | -4.79 | 66.71 | .13 | 2.28 | | 24.03 (<.01) | |  |
| Mixed | 6 | -0.50 | -0.75 to -0.26 | -4.07 | 55.58 |  | 3.62 | | 11.01 (0.05) | |  |
| Treatment modality |  |  |  |  |  |  |  | |  | |  |
| CBT | 10 | -0.62 | -0.86 to -0.38 | -5.10 | 62.01 | .74 | 2.96 | | 23.69 (<.01) | |  |
| CFT | 1 | -0.31 | -1.03 to 0.42 | -0.82 | <.001 |  | 5.75 | | <.01 (>.99) | |  |
| DBT | 1 | -0.84 | -1.03 to -0.32 | -3.17 | <.001 |  | 2.23 | | <.01 (>.99) | |  |
| Dissonance-based | 1 | -0.59 | -1.03 to -0.15 | -2.63 | <.001 |  | 3.09 | | <.01 (>.99) | |  |
| Mixed | 2 | -1.36 | -3.29 to 0.57 | -1.38 | 93.67 |  | 1.51 | | 15.81 (< .01) | |  |
| Format of intervention |  |  |  |  |  |  |  | |  | |  |
| Bibliotherapy | 8 | -0.93 | -1.28 to -0.58 | -5.17 | 65.06 | <.01** | 2.04 | | 20.03 (<.01) | |  |
| CD-ROM | 2 | -0.12 | -0.45 to 0.21 | -0.71 | <.001 |  | 14.71 | | <.01 (>.99) | |  |
| Online | 5 | -0.52 | -0.69 to -0.35 | -5.95 | <.001 |  |  | | <.01 (>.99) | |  |
| Provision of guidance |  |  |  |  |  |  |  | |  | |  |
| Guided | 9 | -0.69 | -0.90 to -0.49 | -6.56 | 48.25 | .93 | 2.67 | | 15.46 (0.05) | |  |
| Unguided | 6 | -0.67 | -1.21 to -0.13 | -22.43 | 80.31 |  | 2.75 | | 25.39 (<.01) | |  |
| Type of guidance |  |  |  |  |  |  |  | |  | |  |
| Email | 3 | -0.82 | -1.09 to -0.54 | -5.90 | <.001 | .04* | 2.28 | | 0.46 (0.80) | |  |
| Online | 2 | -0.39 | -0.61 to -0.16 | -3.37 | <.001 |  | 4.59 | | 0.03 (0.86) | |  |
| Telephone | 2 | -0.57 | -1.61 to -0.49 | -3.69 | 51.62 |  | 3.18 | | 2.07 (0.15) | |  |
| Unknown | 2 | -1.05 | -1.61 to -0.49 | -3.69 | 58.07 |  | 1.85 | | 2.39 (0.12) | |  |
| Qualification of guide |  |  |  |  |  |  |  | |  | |  |
| Eating disorder/CBT specialist (or equivalent) | 1 | -0.83 | -1.29 to -0.36 | -3.49 | <.001 | .87 | 2.26 | | <.01 (>.99) | |  |
| Mental health specialist | 1 | -0.70 | -1.16 to -0.23 | -2.94 | <.001 |  |  | | <.01 (>.99) | |  |
| Non-specialist | 7 | -0.68 | -0.94 to -0.42 | -5.10 | 59.13 |  |  | | 14.68 (0.02) | |  |
|  | | | | | | | | | | |  |
|  |  |  |  |  |  |  |  | |  | |  |
| DSM severity specifier (*g*) | 14 | -0.60 | -0.74 to -0.45 | -8.05 | <.001 | <.01** | 3.09 | | 8.77 (0.79) | |  |
| *Study characteristics* |  |  |  |  |  |  |  | |  | |  |
| Type of eating disorder |  |  |  |  |  |  |  | |  | |  |
| BED | 12 | -0.61 | -0.77 to -0.45 | -7.38 | <.001 | .82 | 2.99 | | 6.05 (0.87) | |  |
| Mixed | 2 | -0.55 | -1.07 to -0.03 | -2.06 | 60.89 |  | 3.31 | | 2.56 (0.11) | |  |
| Treatment modality |  |  |  |  |  |  |  | |  | |  |
| CBT | 11 | -0.56 | -0.72 to -0.40 | -6.93 | <.001 | .48 | 3.25 | | 6.31 (0.79) | |  |
| CFT | 1 | -1.12 | -1.90 to -0.33 | -2.79 | <.001 |  | 1.75 | | <.01 (>.99) | |  |
| DBT | 1 | -0.62 | -1.14 to -0.11 | -2.39 | <.001 |  | 2.96 | | <.01 (>.99) | |  |
| Mixed | 1 | -0.86 | -1.56 to -0.16 | -2.41 | <.001 |  | 1.51 | | <.01 (>.99) | |  |
| Format of intervention |  |  |  |  |  |  |  | |  | |  |
| Bibliotherapy | 9 | -0.65 | -0.84 to -0.47 | -6.91 | <.001 | .65 | 2.82 | | 5.79 (0.67) | |  |
| CD-ROM | 1 | -0.49 | -1.08 to 0.10 | -1.64 | <.001 |  | 3.68 | | <.01 (>.99) | |  |
| Online | 4 | -0.51 | -0.78 to -0.26 | -3.91 | <.001 |  |  | | <.01 (>.99) | |  |
| Provision of guidance |  |  |  |  |  |  |  | |  | |  |
| Guided | 10 | -0.58 | -0.74 to -0.42 | -7.10 | <.001 | .57 | 3.14 | | 3.72 (0.93) | |  |
| Unguided | 4 | -0.72 | -1.16 to -0.27 | -3.17 | 37.95 |  | 2.56 | | 4.84 (0.18) | |  |
| Type of guidance |  |  |  |  |  |  |  | |  | |  |
| Email | 3 | -0.54 | -0.84 to -0.24 | -3.51 | 21.80 | .93 | 3.36 | | 2.56 (0.28) | |  |
| Online | 2 | -0.70 | -1.13 to -0.27 | -3.21 | <.001 |  | 2.63 | | 0.37 (0.54) | |  |
| Telephone | 3 | -0.61 | -0.94 to -0.28 | -3.63 | <.001 |  | 2.99 | | 0.26 (0.88) | |  |
| Unknown | 2 | -0.55 | -0.87 to -0.24 | -3.44 | <.001 |  | 3.31 | | 0.05 (0.83) | |  |
| Qualification of guide |  |  |  |  |  |  |  | |  | |  |
| Eating disorder/CBT specialist (or equivalent) | 1 | -0.29 | -0.74 to 0.16 | -1.26 | <.001 | .39 | 6.17 | | <.01 (>.99) | |  |
| Mental health specialist | 3 | -0.62 | -0.94 to -0.31 | -3.90 | <.001 |  | 2.96 | | 0.66 (0.72) | |  |
| Non-specialist | 6 | -0.63 | -0.83 to -0.42 | -5.97 | <.001 |  | 2.91 | | 1.16 (0.95) | |  |
|  |  |  |  |  |  |  |  | |  | |  |
| Remission/recovery (RR) | 11 | 3.01 | 1.93 to 4.69 | 4.87 | <.001 | <.01** |  | | 41.88 (<.01) | |  |
| *Study characteristics* |  |  |  |  |  |  |  | |  | |  |
| Type of eating disorder |  |  |  |  |  |  |  | |  | |  |
| BED | 5 | 3.77 | 2.06 to 6.87 | 4.32 | <.001 | .28 |  | | 3.37 (0.50) | |  |
| Mixed | 6 | 2.30 | 1.20 to 4.44 | 2.49 | <.001 |  |  | | 3.00 (0.70) | |  |
| Treatment modality |  |  |  |  |  |  |  | |  | |  |
| CBT | 10 | 2.80 | 1.77 to 4.41 | 4.43 | <.001 | .16 |  | | 5.56 (0.78) | |  |
| DBT | 1 | 12.00 | 1.66 to 86.59 | 2.46 | <.001 |  |  | | <.01 (> .99) | |  |
| Format of intervention |  |  |  |  |  |  |  | |  | |  |
| Bibliotherapy | 7 | 3.45 | 1.92 to 6.22 | 4.13 | <.001 | .32 |  | | 4.56 (0.60) | |  |
| CD-ROM | 2 | 1.58 | 0.61 to 4.11 | 0.94 | <.001 |  |  | | 0.64 (0.42) | |  |
| Online | 2 | 3.97 | 1.54 to 10.23 | 2.85 | <.001 |  |  | | 0.06 (0.80) | |  |
| Provision of guidance |  |  |  |  |  |  |  | |  | |  |
| Guided | 10 | 3.62 | 2.21 to 5.93 | 5.11 | <.001 | .09 |  | | 4.73 (0.86) | |  |
| Unguided | 1 | 1.39 | 0.51 to 3.80 | 0.63 | <.001 |  |  | | <.01 (>.99) | |  |
| Type of guidance |  |  |  |  |  |  |  | |  | |  |
| Email | 2 | 3.97 | 1.54 to 10.23 | 2.85 | <.001 | .24 |  | | 0.06 (0.80) | |  |
| Face-to-face | 1 | 7.23 | 0.40 to 134.21 | 1.33 | <.001 |  |  | | <.01 (> .99) | |  |
| Telephone | 4 | 9.25 | 2.92 to 29.37 | 3.78 | <.001 |  |  | | 0.23 0.97) | |  |
| Unknown | 3 | 2.39 | 1.20 to 2.74 | 2.49 | <.001 |  |  | | 0.24 (0.88) | |  |
| Qualification of guide |  |  |  |  |  |  |  | |  | |  |
| Eating disorder/CBT specialist (or equivalent) | 4 | 4.79 | 1.49 to 15.44 | 2.63 | <.001 | .85 |  | | 0.51 (0.92) | |  |
| Mental health specialist | 2 | 3.16 | 1.33 to 7.50 | 2.61 | <.001 |  |  | | 0.61 (0.43) | |  |
| Non-specialist | 4 | 3.89 | 1.76 to 8.61 | 3.53 | 8.87 |  |  | | 3.29 (0.35) | |  |
| *Note.* For hedges’ *g,* negative values favour low intensity psychological intervention. For risk ratio, values > 1 favour low intensity psychological intervention.  BED = Binge Eating Disorder. CBT = Cognitive Behavioural Therapy; DBT = Dialectical Behaviour Therapy; ES = Effect Size; Ncomp = Number of comparisons; NNT = Number Needed to Treat.  * *p* ≤ .05; ***p* ≤ .01. | | | | | | | | | | |  |

**Figure AF6.3** *Funnel plot with imputed studies for studies comparing low intensity psychological interventions against waiting list controls on (1) eating disorder psychopathology; (2) DSM severity specifier-related outcomes; and (3) rates of remission/recovery*


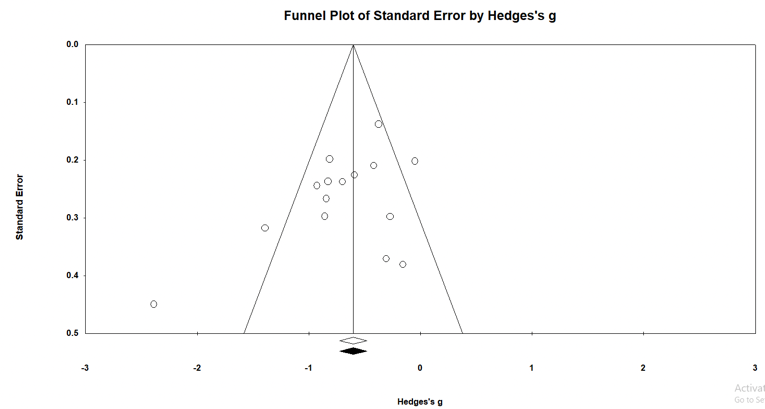

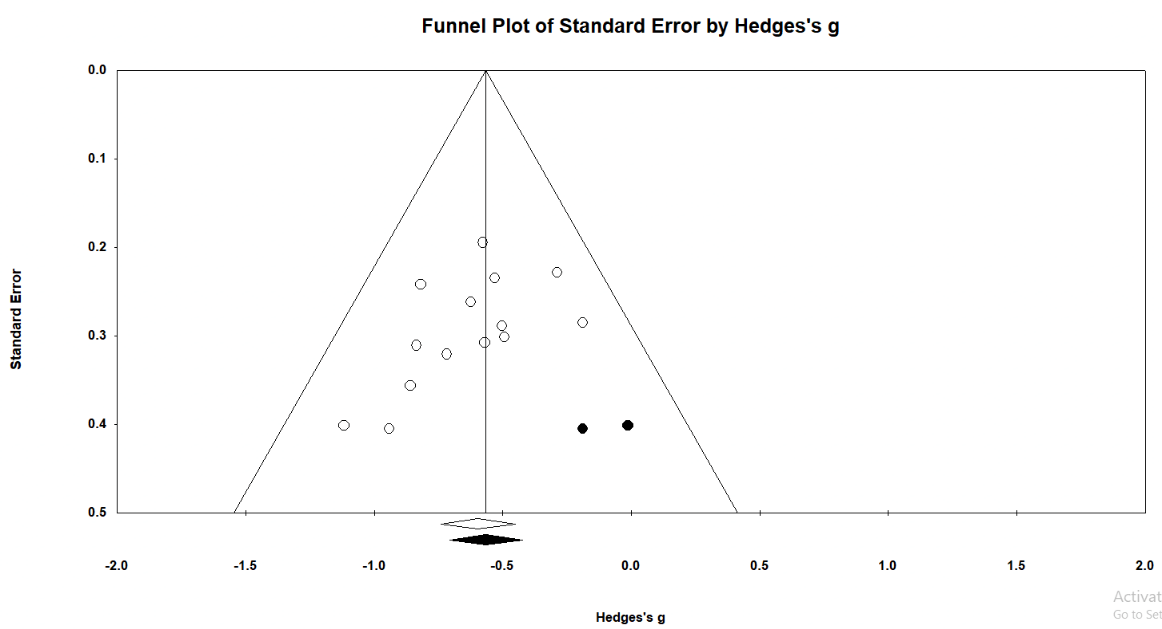

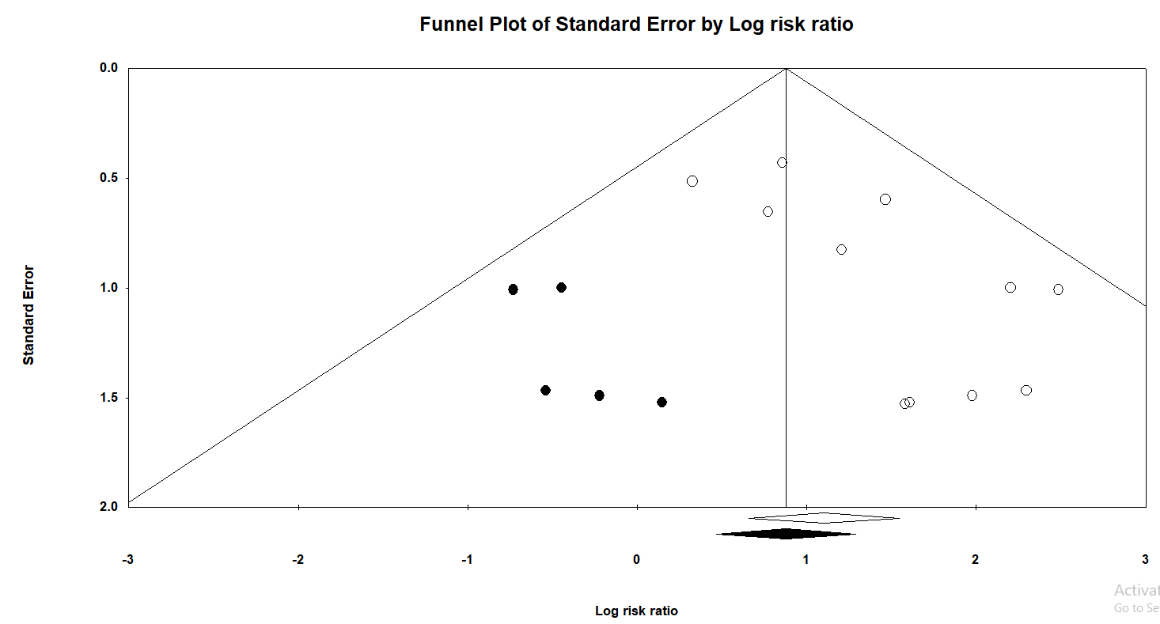


**(1)**

**(2)**

**(3)**
